# Physiological mechanism of lodging resistance of oat stalk and analysis of transcriptome differences

**DOI:** 10.3389/fpls.2025.1532216

**Published:** 2025-04-02

**Authors:** Jiangdi Yu, Ran Zhang, Xiang Ma, Zhifeng Jia, Xiaoxia Li, Yuzhu Li, Huan Liu

**Affiliations:** ^1^ Institute of Ecological Protection and Restoration, Chinese Academy of Forestry, Beijing, China; ^2^ Academy of Animal Sciences and Veterinary, Qinghai University, Xining, China; ^3^ Key Laboratory of Grassland Ecosystem, College of Pratacultural Science, Gansu Agricultural University, Lanzhou, China

**Keywords:** lodging-resistant, transcriptome sequencing, differentially expressed genes, weighted gene co-expression network analysis (WGCNA), Avena sativa

## Abstract

**Introduction:**

Lodging has become an important factor limiting oats production.

**Methods:**

To understand the relationship between oat lodging and stem growth, we selected three oat cultivars with different lodging resistance traits and conducted a detailed analysis of their stem physicochemical properties, and transcriptome sequencing on them at different growth stages were performed.

**Results:**

Important plant characteristics like: length of the second stem internode, stem wall thickness, breaking force, mechanical strength, soluble sugar, starch, lignin, and silicon content were closely related to oat lodging performance. With the growth of the second stem internode at the base of oats, the number of coexpressed differentially expressed genes (DEGs) increased. And DEGs were specifically enriched in starch and sucrose metabolism, phenylpropanoidbiosynthesis, MAPK signaling pathway-plant and carbon metabolism. There were many TF family types among the different comparison groups, and p450, Myb_DNA-binding, WRKY, and AP2 families accounted for the most. Additionally, there was a specific high expression of genes related to the synthesis of cellulose(CesA9, CesA7, and CesA4) and lignin (CCR1, 4CL8, and 4CL3) in lodgingresistant cultivar and middle lodging-resistant cultivar. WGCNA analysis identified genes closely related to lodging resistance, namely MBF1c, SKP1, and CAND1, which were specifically up-regulated on the 35th day of growth in the second stem internode of the highly resistant ‘LENA’. These genes can all serve as positive regulatory factors for oat lodging.

**Discussion:**

Ultimately, our work analyzed the transcriptional network regulatory relationships, laying the foundation for elucidating the physiological and genetic mechanisms of oat lodging resistance, and providing excellent genetic resources for oat and other crop breeding.

## 1 Introduction

Oat (*Avena sativa*) is an annual *Avena* crop from the Gramineae family that is known for its excellent characteristics, such as drought resistance, barren tolerance, saline-alkali tolerance, and rich nutritional value (Singh et al., 2013; Nan, 2021). It has become one of the most important forage crops in the world. In particular, its straw is used as a high-quality forage grass for developing animal husbandry and raising livestock (Ahmad et al., 2014). In recent years, with the increasing development of grassland supporting construction in pastoral areas, the planting area of oat forage base has been expanding, and it has become the dominant forage species for farmers and herdsmen in Qinghai-Tibet Plateau. It is also a leading crop of forage production base and “disaster resistance and livestock protection” high-quality forage in high-altitude pastoral areas, which plays an important role in the stable development of grassland animal husbandry on the Qinghai-Tibet Plateau. However, due to the frequent rainy and windy weather after heading, oats lodge in a large area, affecting the mechanized harvest of forage fields and reducing the yield and quality of forage grass, which has become the main factor affecting the high yield and quality of oats. It is also one of the key problems to be solved in the genetic improvement of oats. Crop lodging is the most common natural phenomenon in the late growth and development of cereal crops, which mainly refers to the phenomenon where stems go from a natural upright state to a permanent dislocation that is caused by the interaction of many factors and is limiting factor in crop production (Shah et al., 2017). Cloninger et al. (1970) classified crops into two categories based on the location of lodging: stem lodging and root lodging. Oat lodging is mainly stem lodging, which is consistent with the research results on lodging of wheat (*Triticum aestivum*). Lodging mainly occurs in the second internode of the base, and the internal causes of lodging are stem dysplasia and reduced mechanical strength of the stem in the internode (Berry and Spink, 2012).

Additionally, plant morphology and microscopic characteristics played an important role in lodging resistance, especially the biological characteristics of root, stem and ear (Silveira et al., 2022). Indeed, the mechanical properties of stem, carbohydrate content and anabolism of structural substances are also factors that determine the lodging resistance of oat (Nan, 2021). In particular, cellulose and lignin are the main components of stem cell wall that played an important role in strengthening it, while also increasing stem compressive strength and bending properties, which can provide mechanical support for the whole culm. Previous research has shown that changes in cellulose and lignin content are closely related to the lodging resistance of oats (Baucher et al., 1998). For instance, Ookawa and Ishihara (1993) found that the cellulose and lignin contents from the stems of lodging-resistant rice (*Oryza sativa*) were higher than those of lodging-susceptible cultivar. Nan (2021) also found that a major contributor for the decrease in stem bending resistance and mechanical strength of oats was low lignin content in the basal internode I and II. It has also been shown that the occurrence of plant lodging is closely related to the growth characteristics of the stem internodes and their physicochemical components.

In recent years, with the continuous improvement of biological technologies, researchers have tried to analyze the regulatory genes related to lodging resistance of oat at the molecular level. At present, many genes coding for enzymes involved in cellulose and lignin biosynthesis pathways related to plant lodging have been cloned and basic functional studies have been carried out. For example, cellulose is synthesized by the cellulose synthase (CesA) complex embedded in the plasma membrane that is connected to form microfibers through hydrogen bonds and van der Waals forces along the microtubule direction and comprises the main polysaccharide in the cell wall. Zhao et al. (2012) found that the activity of cellulose synthase (CesA) is closely related to plant cellulose content. Specifically, *CesA* is a supergene family that participates in many stages of plant cell wall development. For *Arabidopsis thaliana*, *AtCesA1*,-*A3* and-*A6* play a role in the synthesis of the primary cell wall, while *AtCesA4*,-*A7* and-*A8* are directly related to the formation of the secondary cell wall. Yan et al. (2007) found that cellulose content decreases in rice brittle culm. Here, they used a *Brittle Culm* (BC) mutant (*BC1*,-*3*,-*5*,-*7*,-*10*) and barley (*Hordeum vulgare*), and found the brittle culm mutant showed abnormal *CesA* gene expression with a missense mutation. The missense mutation of the secondary cell wall cellulose synthase monomers *OsCESA9* and *OsCESA4* in rice directly led to the hindrance and decrease of the cellulose synthesis in the secondary cell wall, and the stem was easy to break. Taken together, these genes control the content and structure of stem secondary cell wall components, and can make the secondary wall of thick-walled cells thin or loose in structure, reduce the bending stress of the stem, and make the stem brittle and easy to breakage. Lignin also enhances the toughness and mechanical supporting ability of cell walls, and its content is closely related to the mechanical strength and lodging resistance of the stem. Phenylalanine ammonia-lyase (*PAL*), cinnamate 4-hydroxylase (*C4H*), 4-coumarate:CoA lig-ase (*4CL*), cinnamoyl-CoA reductase (*CCR1*/*2*), and caffeic acid O-methyl-transferase (*COMT*) are the main genes coding for enzymes in the lignin biosynthesis pathway. Its expression is closely related to the synthesis and accumulation of lignin, which can effectively regulate the lodging resistance of crops (Liu et al., 2019). Yan et al. (2021) identified the dwarf gene (*Dwarf*) that regulates root and stem growth in oats and developed corresponding molecular markers that can effectively reduce the risk of oat lodging.

In addition, plant lodging resistance is also regulated by a complex transcription factor network (Wei and Lan, 2022). For instance, Ohashi-Ito and Fukuda (2010) found in *Arabidopsis* that the NAC family vascular bundle related NAC-domain 6 and 7 (VND6 and-7) induce vascular bundle cells to transfer to xylem tissue. The NST1, -2 and NST3/SND1 subfamily have also been shown to be key regulators of secondary cell wall formation in xylem tissue. Zhou et al. (2009) found that the overexpression of AtMYB58 and AtMYB63 can specifically activate lignin biosynthesis genes, accompanied by ectopic deposition of lignin in normal unamplified cells. However, others have found that the loss-of-function mutations in AtMYB75 can up-regulated the expression levels of lignin biosynthesis and cellulose-related genes (Bhargava et al., 2010). Wang et al. (2010) also found that mutations in WRKY family increased the biosynthesis of xylan, cellulose, and lignin, which are needed for secondary wall thickening, and result in a 50% increase in stem biomass. To sum up, these genes and TFs affect the lodging resistance of plant stems by regulating many physicochemical properties.

At present, it has been recognized that the morphological characteristics, physicochemical properties, and gene expression regulation of stem internodes have important effects on crop stem mechanical strength and lodging resistance, but their specific relationship and influence mechanism are still unclear. In alpine pastoral areas, culm lodging mainly occurs during oat planting, a transcriptional study on lodging resistance of oat has not been reported, therefore, it is particularly necessary to study the mechanism of oat resistance to lodging. Our study selected oat cultivars with different lodging resistance to investigate physiological indicators, such as stem morphology and physicochemical properties, as well as comparative transcriptome data, to accurately locate genes related to oat stem strength and lodging resistance. Our goal was to understand the molecular basis of functional performance for lodging-resistant cultivars and their related main control genes are expressed. Here, we show a network of regulatory relationships at the transcriptional level that will provide basic data to control the genetic maintenance of excellent traits at the gene expression level for lodging-resistant species and accelerate the development of animal husbandry in alpine pastoral areas.

## 2 Materials and methods

### 2.1 Plants materials

Based on previous research (Zhang et al., 2020), three oat cultivars, ‘LENA’ (*Avena sativa* L. cv. LENA, high lodging-resistant cultivar, LN), ‘Qingyin No.1’ (middle lodging-resistant cultivar, QH) and ‘Qingyin No.2’ (lodging-susceptible cultivar, QY), were sown in Lusha Town, Huangzhong County, Qinghai Province, Qinghai Tibet Plateau, China in April 2023 and 2024, respectively. The geographical location was 101°37’ E, 36°28’ N, with an elevation of 2620 m, average annual precipitation 481 mm, and is characterized as a flat terrain with good fertility. The selection of materials is based on field experiments from the past ten years.

### 2.2 Experimental design

A single factor random block design was used for experiments. Each material was sown in a plot with a length of 5 m, a width of 3 m, and an area of 5 m × 3 m = 15 m^2^. Strip planting was used with a sowing rate of 225 kg·hm^2^, a depth of 3-4 cm, and at a rate of 3.75 million stems per hectare.

### 2.3 Determination of field characteristics

During the flowering stage, 10 plants were randomly selected from each plot of the three oat cultivars. Their plant, center of gravity, and ear height were recorded with a tape measure. Here, ear position coefficient = ear height/plant height. The fresh weight of each plant was weighed immediately after root removal. The second stem internode length and internode below the panicle length were measured with a ruler.

The incidence of lodging was determined and the actual area of lodging measured at flowering and before the harvest stage. The actual lodging rate is calculated using the following formula:


Actual lodging rate=actual lodging area/plot area ×100%

### 2.4 Determination of mechanical properties and physiological indicators related to oat lodging

Randomly select 10 plants from each plot of three oat cultivars. The length from the base of the second stem internode to the top of the ear and the weight from the base of the second stem internode to the top of the ear were measured with a tape measure and balance. The stem diameter and wall thickness of the second stem internode were measured with a vernier caliper. The compression strength, puncture strength, breaking force, and mechanical strength of the second stem internode were measured by plant stem strength tester (YYD-1) (Wang et al., 2019). The bending moment is calculated using the following formula:

Bending moment = the fresh weight from the base of the internode to the top of the ear × the length from the base of the internode to the top of the ear, in g·cm^-1^ (Lei et al., 2013).

The collected samples were processed by removing the ears, leaves and roots. The stems were blanched at 105°C for 30 min and then dried at 65°C for 48 h, and then crushed. The contents of lignin, soluble sugar and starch, as well as the contents of calcium, potassium and silicon were determined using the Solabio kit (Beijing, China). For each index, 0.05~0.1 g was weighed, with three biological replicates. The specific steps were followed as per the instructions.

### 2.5 Comparative transcriptome analysis of different lodging-resistant oat stems

#### 2.5.1 Illumina sequencing and data analysis

Samples were collected on the 21st, 28th, and 35th day after the growth of the second stem internode at the base of oats, and three biological repeats were set at each time point. Extracted sample RNA and Illumina’s NEB Next^®^ Ultra™ RNA Library Prep Kit for Illumina^®^ kit were used to build a cDNA library for testing. After passing library inspection, different libraries were sequenced by the Illumina NovaSeq 6000 after pooling, according to the effective concentration and the target off-machine data demand.

The original image data obtained from the high-throughput sequencing was transformed into raw reads using CASAVA base recognition. Clean reads were filtered and spliced. End reads were aligned using the reference genome (https://plants.ensembl.org/index.html) from HISAT2 v2.0.5 (Mortazavi et al., 2008). After, the samples were analyzed by principal component analysis (PCA).

#### 2.5.2 Screening and enrichment analysis of differentially expressed genes

We used DESeq2 (Love et al., 2014) to analyze differential expression between sample groups. DEGs were obtained based on screening criteria of a difference multiple |log2Fold Change| ≥ 1 and a false discovery rate (FDR)< 0.05. GO enrichment analysis and KEGG enrichment analysis of differentially expressed genes were realized using clusterProfiler (3.8.1) software.

#### 2.5.3 Transcription factor analysis

Plant TF prediction was carried out using iTAK (Zheng et al., 2016) software, which integrates two databases, PlnTFDB and PlantTFDB. Following analysis, it identifies and classifies data according to methods from Pérez-Rodríguez et al. (2010).

#### 2.5.4 DEGs expression clustering analysis

In order to explore the expression trend of DEGs in different growth stages, the scale () function of R was used to standardize all DEGs with union FPKM. The processed DEGs were then analyzed by Kmeans cluster analysis.

#### 2.5.5 Weighted gene co-expression network analysis

Our co-expression network was constructed using the WGCNA package of R (Langfelder and Horvath, 2008), and samples are clustered and analyzed using the methods from Qin et al. (2020). The soft threshold β parameter is calculated by using the pickSoftThreshold function in WGCNA to meet the prerequisite of scale-free network distribution. Select the threshold parameter β when R2 reaches 0.8, calculate the dissimilarity coefficient between all genes to create the adjacency matrix, use the dissimilarity degree dissimilarity between genes to cluster the genes in the network, and establish a hierarchical clustering tree, and then use the dynamic cutting method to cut the tree into different modules (the minimum number of genes is 30), and merge the modules whose correlation coefficient are greater than 0.75, those are, the dissimilarity coefficient are less than 0.25 (Wu et al., 2022). Protein-protein interaction (PPI) network was constructed using STRING database. Cytoscape (version 3.8.2) (Shannon et al., 2003) is used to visualize the PPI network, using the plug-in cytoHubba to determine the central gene by Maximal Clique Centrality (MCC) calculation.

### 2.6 Statistical analysis

Analysis of variance (ANOVA) was performed on all data, and Windows version of SPSS 24.0 (SPSS Inc., New York, NY, USA) was used, and the mean separation was performed with Fisher’s protected least significant difference (LSD) test at 0.05 significance level. SPSS 24.0 was used for Pearson correlation analysis.

## 3 Results

### 3.1 Comparative analysis of field traits and stem mechanical characteristics for different oat cultivars

As shown in **Table 1**, ‘Qingyin No.1’ showed the highest plant height, center of gravity height, and ear height in 2023 and 2024, and was significantly higher than ‘Qingyin No.2’ and ‘LENA’ (*P*< 0.05). The mechanical strength of ‘LENA’ was the highest, and was significantly higher than that of ‘Qingyin No.1’ and ‘Qingyin No.2’ (*P*< 0.05). The second stem internode length, center of gravity height coefficient, and ear height of ‘Qingyin No.2’ was the highest, while ‘LENA’ was the lowest, and was significantly lower than the other two cultivars (*P*< 0.05). In 2023, ‘LENA’ had the highest single plant fresh weight, second stem internode stem diameter, stem wall thickness, compression strength, puncture strength, breaking force, and bending moment was significantly higher than the other two cultivars (*P*< 0.05). However, single plant fresh weight was the lowest in ‘LENA’, while ‘Qingyin No.2’ had the lowest value for the other indicators. In 2024, ‘Qingyin No.1’ had the highest single plant fresh weight, second stem internode stem diameter, stem wall thickness, compression strength, puncture strength, breaking force, and bending moment. However, it did not have the lowest second stem internode stem diameter and stem wall thickness. Still, there were no significant differences compared to ‘LENA’, and all other indicators were significantly higher than the other cultivars (*P*< 0.05). Here, ‘Qingyin No.2’ had the lowest for these indicators.

**Table 1:** Comparative analysis of field traits and stem mechanical characteristics in the second stem internode from three different lodging-resistant oat cultivars in 2023 and 2024.

Year	Cultivar	Plant height (cm)	Single plant fresh weight (g)	Second stem internode length (cm)	Stem diameter (mm)	Stem wall thickness (mm)	Compression strength (kg)	Puncture strength (kg)	Breaking force (kg)	Mechanical strength (kg)	Bending moment(g·cm^-1^)	Center of gravity height (cm)	Center of gravity height coefficient	Ear height (cm)	Ear position coefficient
2023	LENA	104.48± 1.46c	12.69± 0.45a	13.85± 0.27c	4.61± 0.11a	0.66± 0.03a	14.98± 0.46a	10.63± 0.65a	12.90± 1.04a	1202± 103.73a	1059± 49.47a	43.10± 0.54c	0.41± 0.004b	85.58± 1.29c	0.82± 0.003c
	Qingyin No.1	128.95± 1.68a	10.97± 0.54b	20.65± 0.33b	3.92± 0.06b	0.44± 0.02b	10.45± 0.68b	8.07± 0.65b	9.48± 0.72b	853± 72.42b	999± 42.32b	57.88± 0.82a	0.45± 0.002a	110.43 ± 1.77a	0.86± 0.04a
	Qingyin No.2	121.83± 1.25b	11.01± 0.69b	22.35± 0.31a	3.75± 0.07b	0.24± 0.02c	8.23± 0.65c	7.99± 0.53b	7.62± 0.57b	515± 57.43c	926± 54.55c	50.01± 0.99b	0.41± 0.006b	102.46 ± 1.29b	0.84± 0.003b
2024	LENA	136.08 ± 2.23b	27.04 ± 1.53b	19.89 ± 0.65b	6.73 ± 0.23a	1.12 ± 0.06a	8.06 ± 0.93b	9.18 ± 0.49b	8.92 ± 0.81b	1323 ± 112.66a	452.50 ± 32.38b	55.86 ± 1.28c	0.41 ± 0.003c	75.63 ± 1.09c	0.56 ± 0.002c
	Qingyin No.1	169.70 ± 2.17a	39.64 ± 3.15a	22.85 ± 1.05a	7.37 ± 0.28a	1.26 ± 0.09a	15.08 ± 1.26a	13.61 ± 1.08a	17.72 ± 1.86a	924 ± 84.35b	730.29 ± 49.36a	70.46 ± 0.90a	0.42 ± 0.006b	109.82 ± 1.65a	0.65 ± 0.004b
	Qingyin No.2	139.63 ± 1.31b	17.49 ± 0.59c	23.91 ± 0.49a	5.45 ± 0.12b	0.73 ± 0.03b	4.89 ± 0.48c	8.59 ± 0.28b	4.33 ± 0.37c	613 ± 53.63c	372.28 ± 27.64c	60.56 ± 1.79b	0.43 ± 0.002a	103.23 ± 1.36b	0.74 ± 0.005a

Different letters in same column means significant differences at *P<* 0.05. The same below.

### 3.2 Analysis of physicochemical properties in stems from different oat cultivars

**Figure 1:**
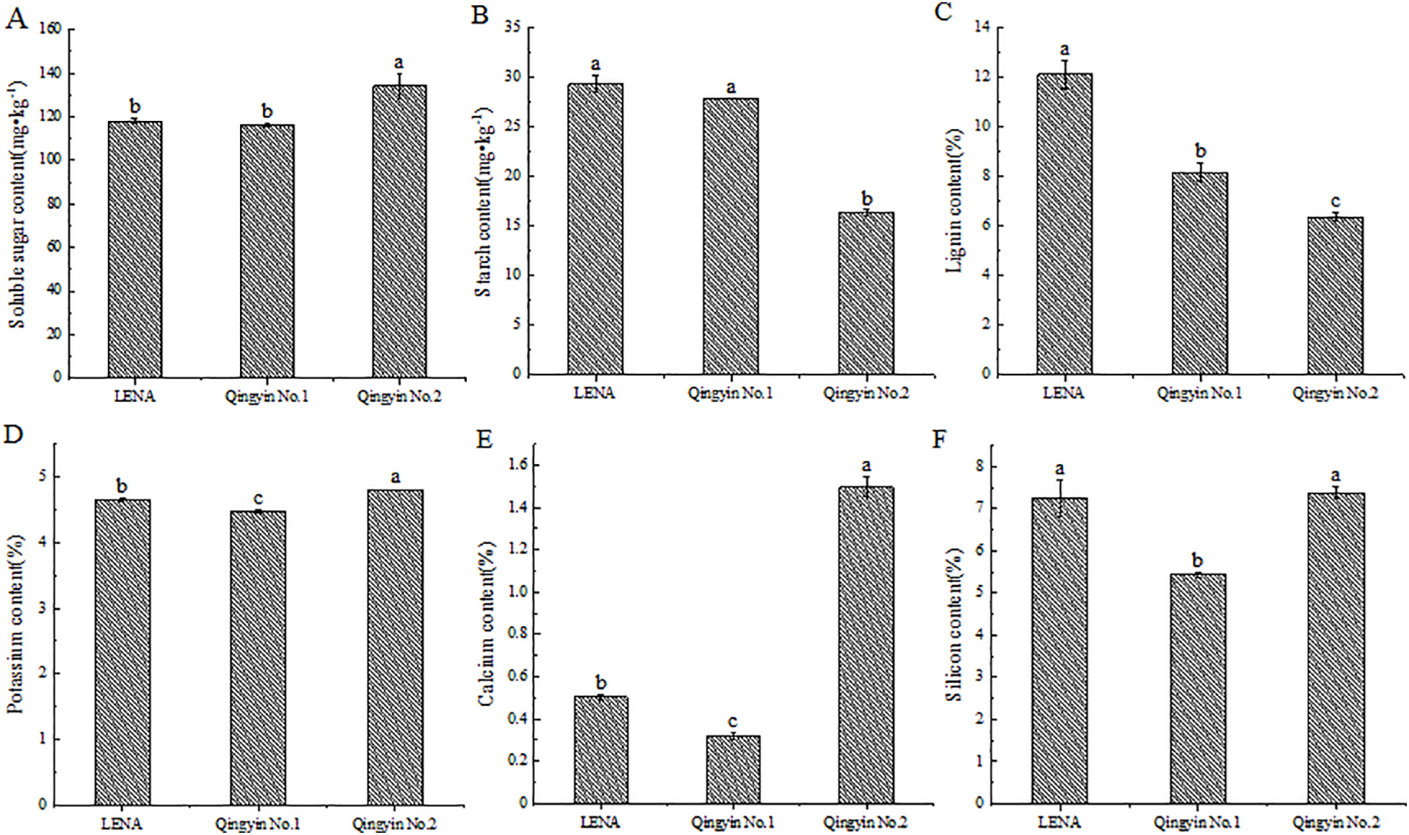
Changes in soluble sugar **(A)**, starch **(B)**, lignin **(C)**, potassium **(D)**, calcium **(E)** and silicon **(F)** contents in stems from different oat cultivars. Different lowercase letters in the figure represent significant difference at the 0.05 level.

We determined that there are differences in the soluble sugar, starch, lignin, potassium, calcium, and silicon content among the different oat cultivars during the flowering period. The soluble sugar, potassium, calcium, and silicon contents from ‘Qingyin No.2’ were the highest, except for the silicon content, which had no significant difference with ‘LENA’. All other indicators were significantly higher than the other two cultivars (*P*< 0.05). ‘LENA’ had the second highest content of these indicators, while ‘Qingyin No.1’ had the lowest content (**Figures 1A, D–F**). The starch content and lignin content in ‘LENA’ were the highest, followed by ‘Qingyin No.1’. There was no significant difference in starch content between ‘LENA’ and ‘Qingyin No.1’ (*P* > 0.05), while ‘Qingyin No.2’ had the lowest content, and was significantly lower than the other two cultivars (*P*< 0.05) (**Figures 1B, C**).

### 3.3 Correlation analysis between field traits, stem mechanical characteristics, physiological indicators, lodging index, and lodging rates in oats

The differences between the lodging index in the second stem internode during the flowering period and the actual lodging rate were extremely significant (*P*< 0.01), and were significantly positively correlated. The higher the value of both, the weaker the lodging resistance. The lodging index and actual lodging rate of the second stem internode were highly significant positively correlated with the second stem internode length (*P*< 0.01). However, it was highly significant negatively correlated with stem wall thickness, compression strength, puncture strength, breaking force, and mechanical strength, as well as soluble sugar, starch, lignin, and silicon content from the second stem internode (*P*< 0.01), and significantly negatively correlated with potassium content (*P<* 0.05). We also found that the lodging index of the second stem internode were significantly positively correlated with plant height, ear height, and ear position coefficient (*P*< 0.05), but were significantly negatively correlated with calcium content (*P*< 0.01). In addition, the actual lodging rate were highly significant negatively correlated with stem wall thickness in the second stem internode stem (*P*< 0.01) and significantly negatively correlated with calcium content (*P*< 0.05) (**Table 2**).

**Table 2:** Correlation analysis between field traits, stem mechanical characteristics, physiological indicators, lodging index, and lodging rate in oats.

	X1	X2	X3	X4	X5	X6	X7	X8	X9	X10	X11	X12	X13	X14	X15	X16	X17	X18	X19	X20	L1	L2
L1	0.498*	-0.076	0.790**	-0.396	-0.835**	-0.810**	-0.703**	-0.644**	-0.657**	-0.074	0.389	0.014	0.504*	0.442*	-0.793**	-0.677**	-0.772**	-0.699*	-0.876**	-0.819**	1	0.915**
L2	0.202	-0.360	0.589**	-0.633**	-0.803**	-0.615**	-0.537**	-0.786**	-0.804**	-0.358	0.090	-0.259	0.233	0.380	-0.799**	-0.692**	-0.770**	-0.685*	-0.845*	-0.939**	0.915**	1

X1, Plant height; X2, Single plant fresh weight; X3, Second stem internode length; X4, Stem diameter; X5, Stem wall thickness; X6, Compression strength; X7, Puncture strength; X8, Breaking force; X9, Mechanical strength; X10, Bending moment; X11, Center of gravity height; X12, Center of gravity height coefficient; X13, Ear height; X14, Ear position coefficient; X15, Soluble sugar; X16, Starch; X17, Lignin; X18, Potassium; X19, Calcium; X20, Silicon; L1, Lodging index of second stem internode; L2, Actual lodging rate.

* indicates that the correlation is significant at 0.05 level, and * * correlation coefficient is significant at 0.01 level.

### 3.4 Comparative transcriptome analysis of oat stems with different lodging resistance

#### 3.4.1 Quality analysis of RNA sequencing data

Each filtered clean read had a total of 293.22 Gb, while clean bases were above 9 Gb. The total number of bases G and C as a percentage of the total number of bases (GC content) was 53.87% and 55.75%, respectively, while the reads compared to the reference genome ranged from 94.88% to 97.18% (**Supplementary Table S1**). PCA analysis revealed that the different treatments for the three oat cultivars were clustered together, and the three biological replicates of each cultivar were clustered together (**Supplementary Figure S1**). Together, this indicates the consistency of the biological replicates, as well as the quality of transcriptome sequencing data, which can be used for subsequent biological analysis.

#### 3.4.2 Screening of differentially expressed genes

**Figure 2:**
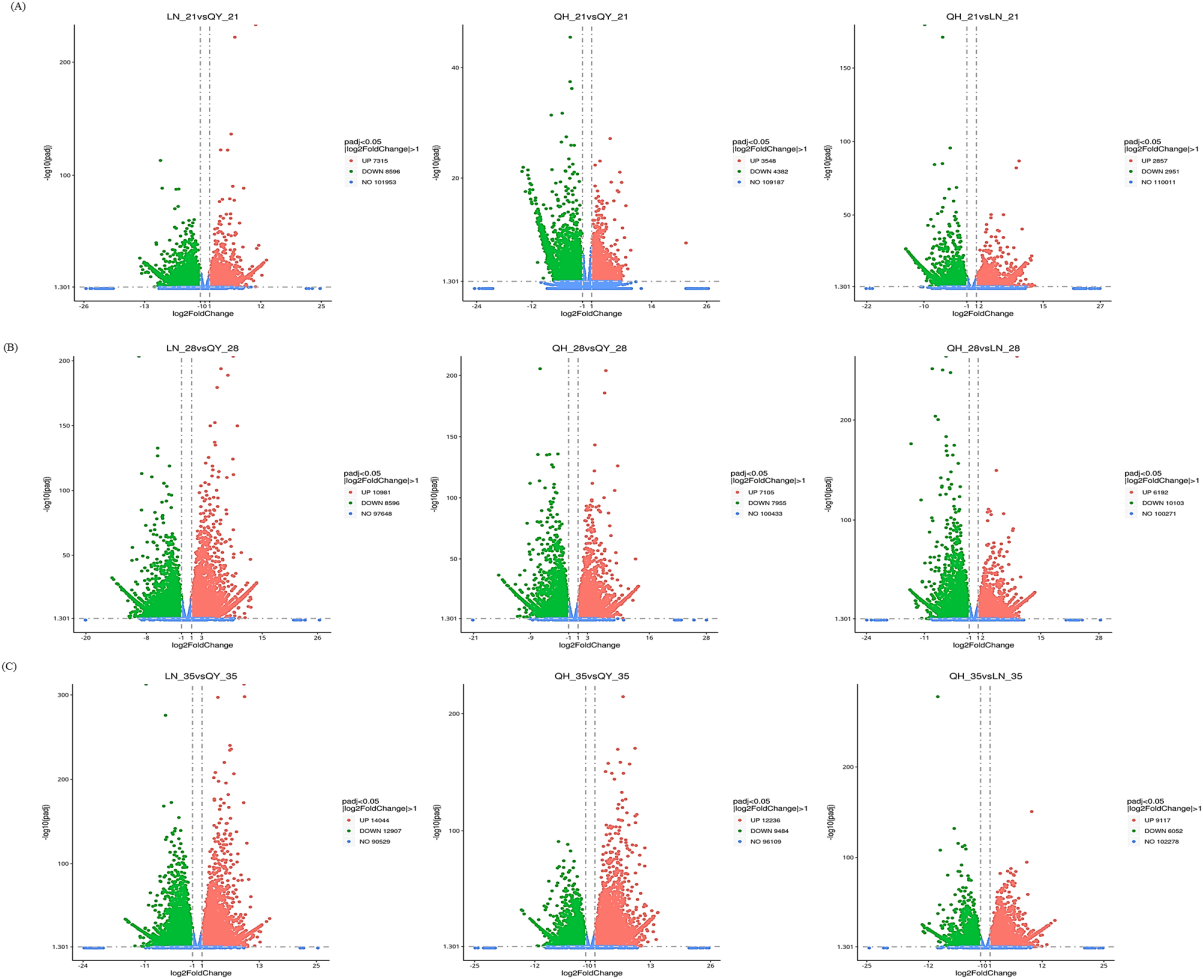
Volcanic diagram analysis of DEGs on the 21st **(A)**, 28th **(B)**, and 35th day **(C)** of growth in the second stem internode at the base for different oat cultivars. Red indicates up-regulated DEGs, green indicates down-regulated DEGs, and blue indicates unexpressed DEGs.

During the growth of oat stems, the expression of many genes were significantly induced or inhibited. LN_35 vs QY_35, QH_35 vs QY_35 and QH_28 vs LN_28 had the most DEGs. This was followed by LN_28 vs QY_28, QH_28 vs QY_28 and QH_35 vs LN_35, respectively (**Figure 2B, C**). The number of DEGs for LN_21 vs QY_21, QH_21 vs QY_21, and QH_21 vs LN_21 was the least (**Figure 2A**). Our results showed that there were more genes involved in oat stem growth, and especially during the later stage of oat stem growth.

**Figure 3:**
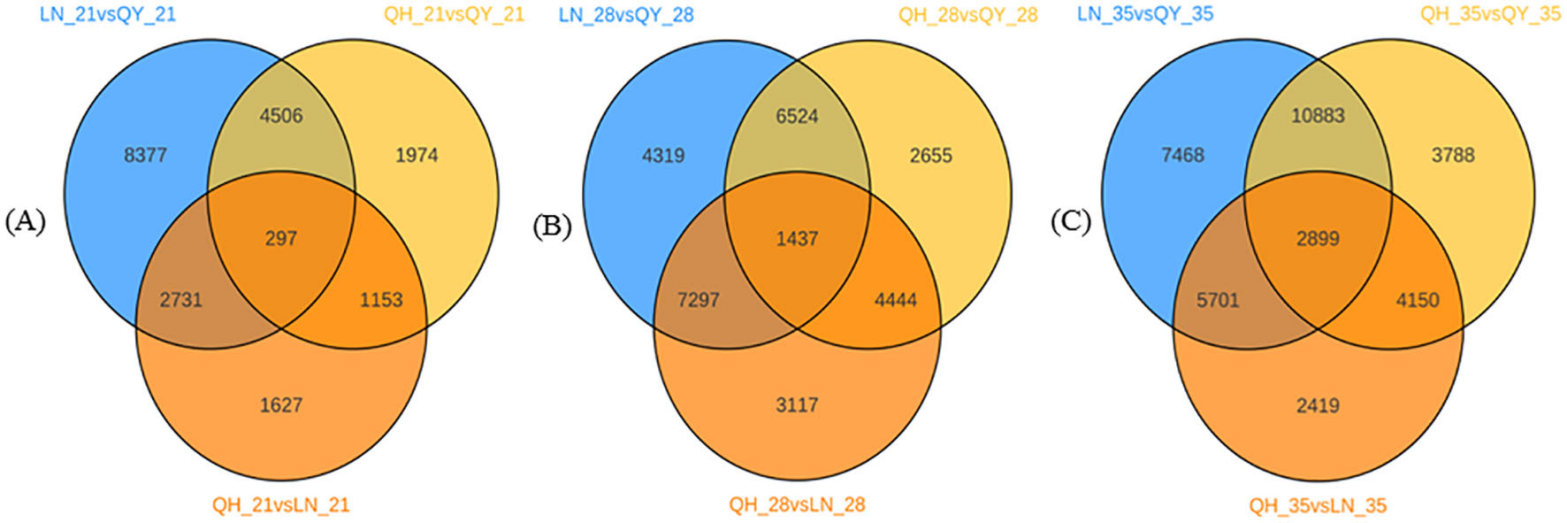
Venn diagram analysis of DEGs on the 21st **(A)**, 28th **(B)**, and 35th day **(C)** of growth in the basal second stem internode for different oat cultivars.

The 21st day of growth at the internode of the second stem at the base, 297 DEGs were co-expressed among the three oat cultivars (**Figure 3A**). 1437 DEGs were co-expressed on the 28th day (**Figure 3B**), and 2899 DEGs were co-expressed on the 35th day (**Figure 3C**). It can be seen that with the growth of the second stem internode at the base of oats, with an increase in co-expressed DEGs, the more complex the expression regulation pathway tends to be.

#### 3.4.3 GO and KEGG enrichment analysis of DEGs

**Figure 4:**
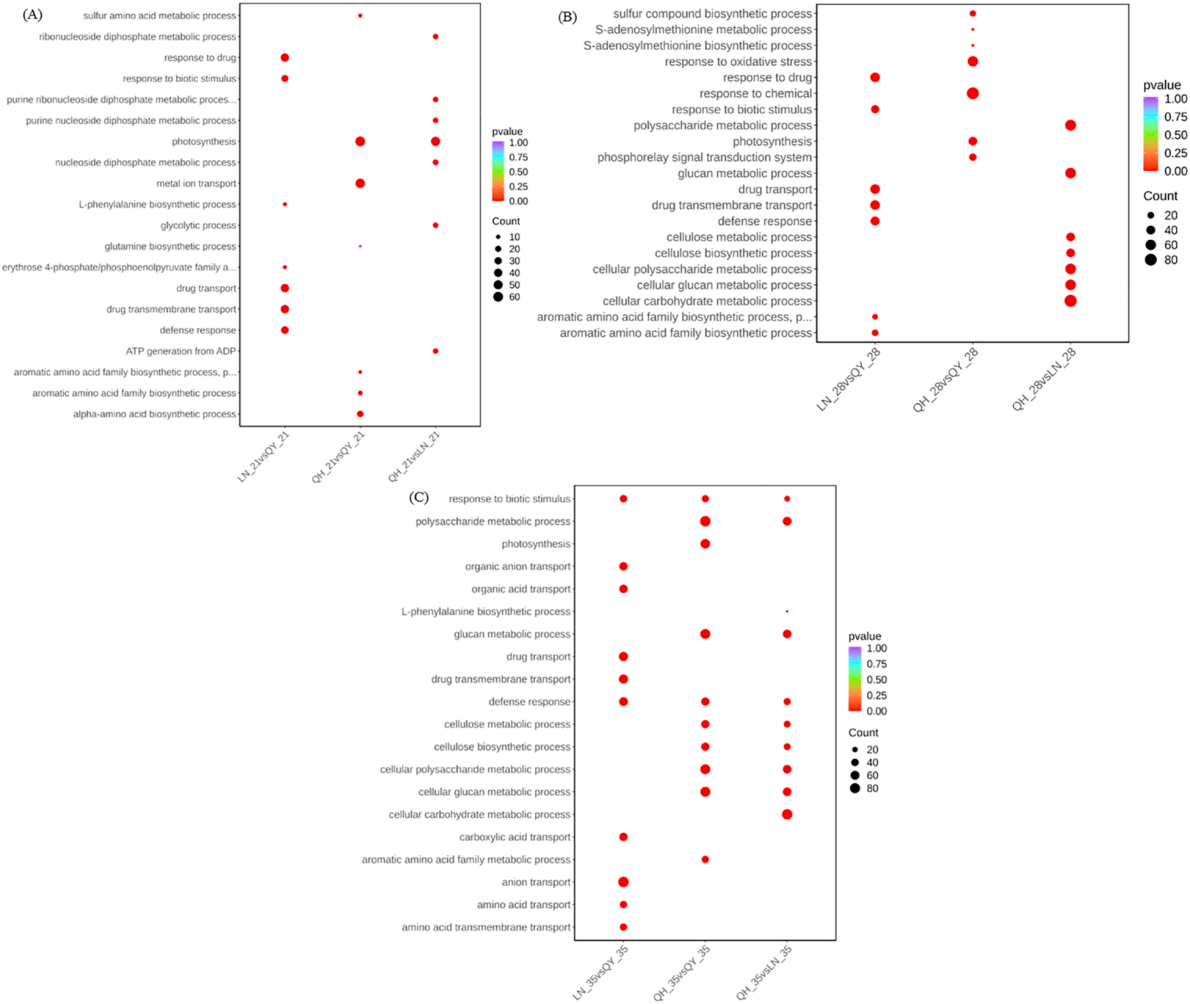
GO enrichment and scatter plots for DEGs on the 21st **(A)**, 28th **(B)** and 35th day **(C)** of growth in the basal second stem internode from different oat cultivars. The smaller the q-value, the higher the enrichment degree. Same as below.

Gene ontology (GO) function enrichment analysis showed that the 21st and 28th days of growth at the internode of the second stem at the base of oat, the DEGs from LN_21 vs QY_21 and LN_28 vs QY_28 were significantly enriched in response to drug, drug transport, drug transmembrane transport, and other pathways (**Figure 4A, B**). Meanwhile, on the 35th day, the DEGs from LN_35 vs QY_35, QH_35 vs QY_35 and QH_35 vs LN_35 were significantly enriched in response to biotic stimulus and defense response. Among them, the DEGs from LN_35 vs QY_35 were specifically enriched in anion transport, organic anion transport, organic acid transport and other pathways (**Figure 4C**).

**Figure 5:**
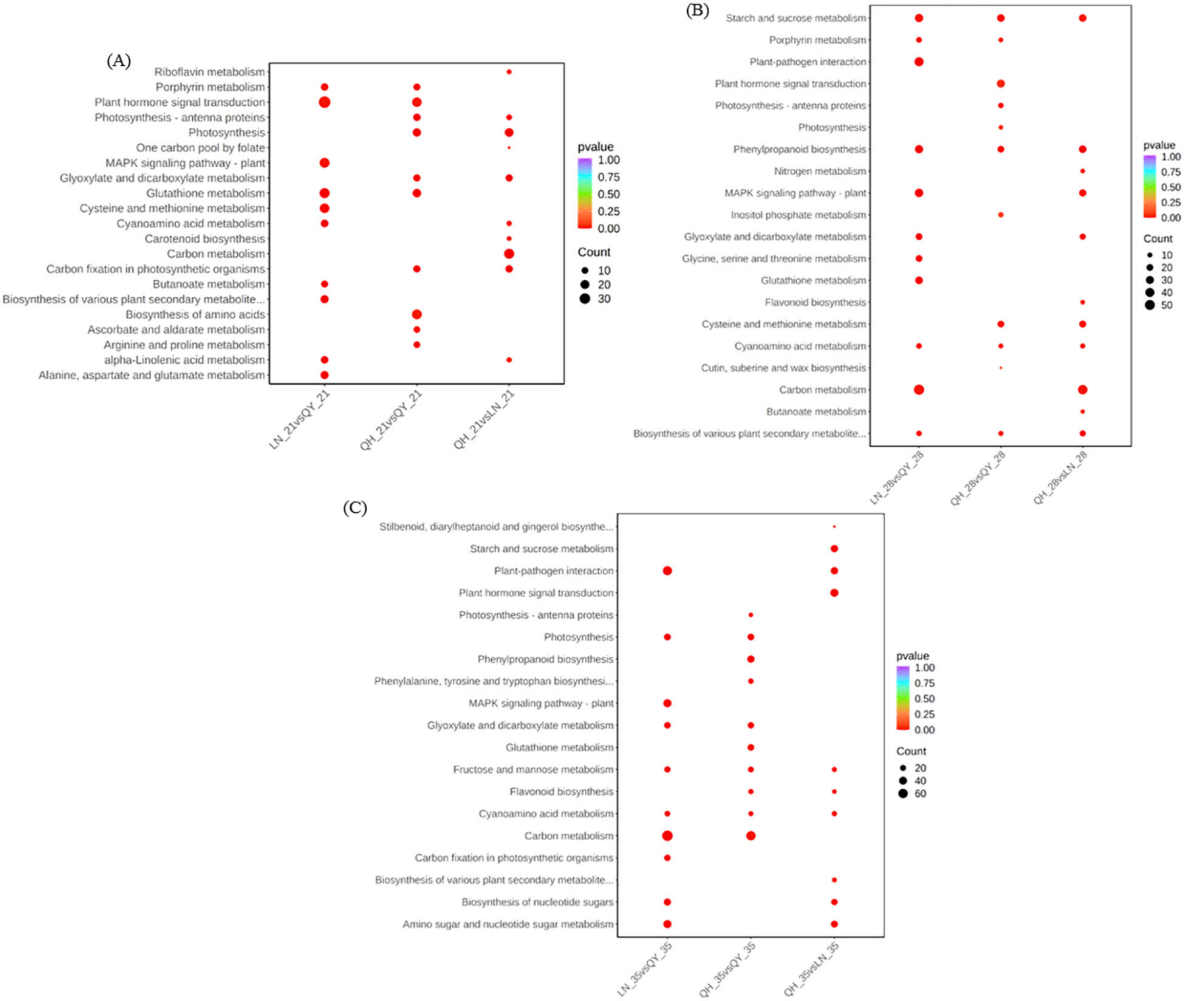
KEGG enrichment scatter plots for DEGs on the 21st **(A)**, 28th **(B)**, and 35th day **(C)** of growth in the second stem internode at the base of different oat cultivars.

In order to further explore the metabolic pathways involved, kyoto encyclopedia of genes and genomes (KEGG) enrichment analysis showed that the DEGs from LN_21 vs QY_21 were significantly enriched in plant hormone signal transduction, MAPK signaling pathway-plant, glutathione metabolism and other pathways on the 21st day after the formation (**Figure 5A**). On the 28th day, the DEGs from LN_28 vs QY_28, QH_28 vs QY_28 and QH_28 vs LN_28 showed abundant pathways in starch and sucrose metabolism, phenylpropanoid biosynthesis, cyanoamino acid metabolism, among them. Meanwhile, the DEGs from LN_28 vs QY_28 were specifically enriched in plant-pathogen interaction, glutathione metabolism, as well as glycine, serine, and threonine metabolism (**Figure 5B**). On the 35th day, the DEGs from LN_35 vs QY_35, QH_35 vs QY_35 and QH_35 vs LN_35 were significantly enriched in fructose and mannose metabolism and cyanoamino acid metabolism. Among them, most DEGs were specifically enriched in starch and sucrose metabolism, phenylpropanoid biosynthesis, MAPK signaling pathway-plant and carbon metabolism (**Figures 5A–C)**. And a large amount of DEGs are enriched in these pathways, indicating that these KEGG pathways are closely related to the lodging resistance of oat stems.

#### 3.4.4 Transcription factor analysis

**Figure 6:**
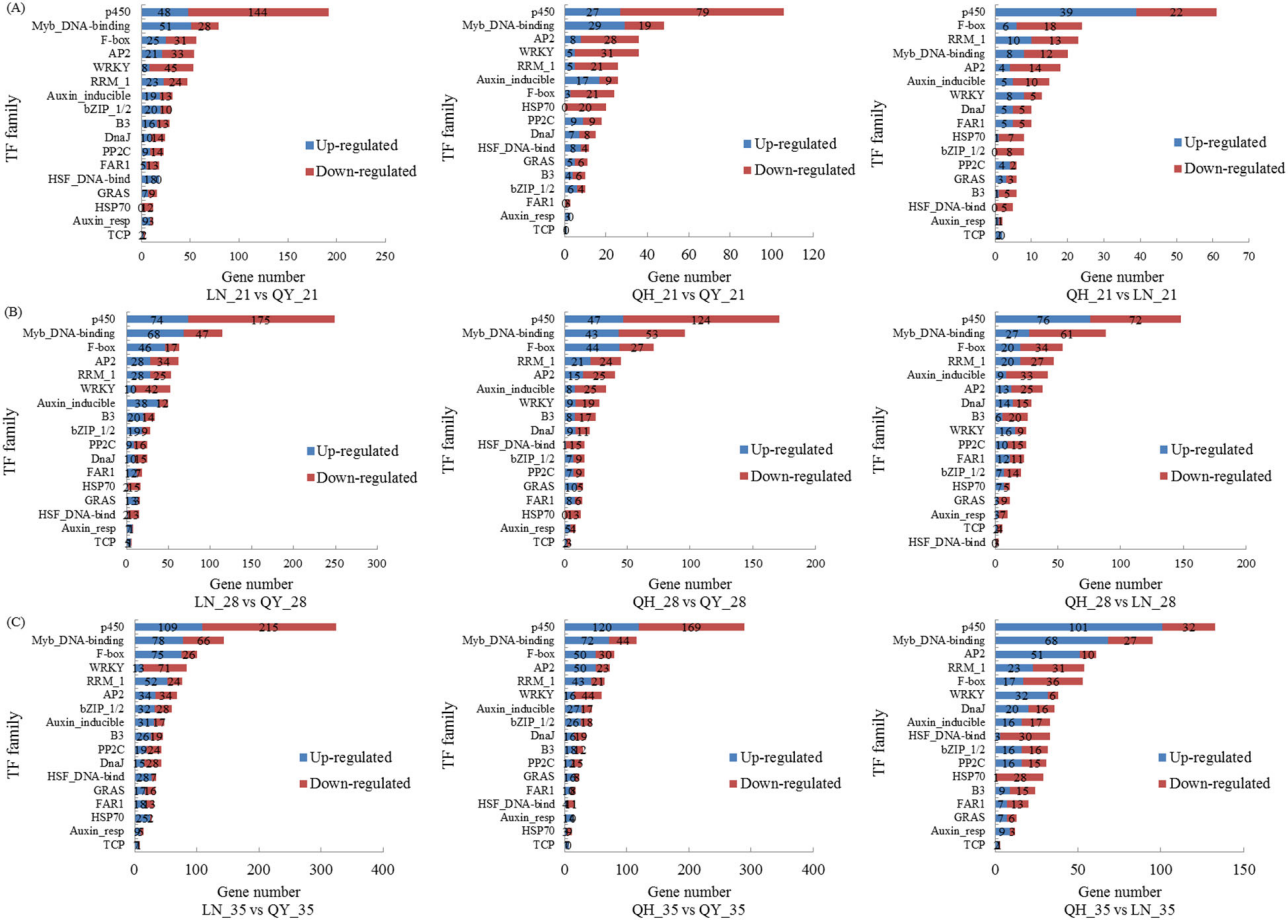
Taxonomic statistics for transcription factors on the 21st **(A)**, 28th **(B)** and 35th day **(C)** of growth in the basal second stem internode of different oat cultivars. Figure shows the 17 TF families with the largest number of DEGs.

Our RNA-seq analysis indicate that many TFs are involved in regulating the growth of oat stems. On the 21st day, the TF families with the highest proportion of differential groups were p450, Myb_DNA-binding, AP2, F-box, and WRKY (**Figure 6A**). On the 28th and 35th days, the TF families with the highest proportion were p450, Myb_DNA-binding, F-box, RRM1, AP2, and WRKY (**Figures 6B, C**). The up-regulated TFs were mainly enriched in the Myb_DNA-binding family, while the down-regulated TFs were mainly found in the p450, WRKY, F-box, RRM1, and AP2 families. There were many TF family types among the different comparison groups, and p450, Myb_DNA-binding, WRKY, and AP2 families accounted for the most.

Based on the number of DEGs, the value of log_2_FoldChange (Log_2_FC), and the level of expression (FPKM), the 35th day comparison group was selected for further analysis of TFs. The results showed that the expression of *MYB86*, *ODORANT1*, *MYBHv33*, *MYB308*, and *MYB39* in the Myb_DNA-binding family; *CYP89A9*, *CYP75A5*, *CYP98A1*, *CYP710A1* and *CYP714B2* in the p450 family; *ERF055*, *DREB3* and *ERF034* in the AP2 family were significantly up-regulated in lodging-resistant cultivar (‘LENA’ and ‘Qingyin No.1’). And the log_2_FC of multiple TFs was more than 3 times that of ‘Qingyin No.2’; the expression of *WRKY71*, *WRKY46*, *WRKY57* and *WRKY6* of the WRKY family were significantly down-regulated compared to the ‘Qingyin No.2’, while *WRKY22* showed up-regulated; The *ERF1A* of the AP2 family were down-regulated in LN_35 vs QY_35 and QH_35 vs QY_35, and up-regulated in QH_35 vs LN_35, the *DREB1H* were down-regulated in LN_35 vs QY_35 and up-regulated in QH_35 vs QY_35 and QH_35 vs LN_35 (**Table 3**).

**Table 3:** Transcriptional factor screening on the 35th day of internode growth of the second stem of different oat cultivars.

Myb_DNA-binding				WRKY				AP2				p450			
TF ID/Name	LN_35vsQY_ 35	QH_35vsQY_ 35	QH_35vsLN_ 35	TF ID/Name	LN_35vsQY_ 35	QH_35vsQY_ 35	QH_35vsLN_ 35	TF ID/Name	LN_35vsQY_ 35	QH_35vsQY_ 35	QH_35vsLN_ 35	TF ID/Name	LN_35vsQY_ 35	QH_35vsQY_ 35	QH_35vsLN_ 35
AVESA.00010b.r2. 7AG1235390 (MYB86)	5.1472 (up)	8.1078 (up)	2.9552 (up)	AVESA.00010b.r2. 3CG0490060 (WRKY6)	-4.6442 (down)	-1.4494 (down)	3.1971 (up)	AVESA.00010b.r2. 7CG0674300 (ERF055)	9.8064 (up)	7.3167 (up)	-2.4868 (down)	AVESA.00010b.r2. 7DG1353800 (CYP89A9)	7.3592 (up)	5.5624 (up)	-1.7906 (down)
AVESA.00010b.r2. 7AG1221470 (ODORANT1)	4.9837 (up)	7.9101 (up)	2.9223 (up)	AVESA.00010b.r2. 7AG1246260 (WRKY22)	3.3798 (up)	1.99968 (up)	-1.3765 (down)	AVESA.00010b.r2. 6CG1103390 (DREB3)	4.6123 (up)	8.2026 (up)	3.5989 (up)	AVESA.00010b.r2. 5DG0998210 (CYP75A5)	7.2544 (up)	8.3773 (up)	1.1235 (up)
AVESA.00010b.r2. 1AG0006970 (MYBHv33)	4.8892 (up)	9.0396 (up)	4.1500 (up)	AVESA.00010b.r2. 4CG1258480 (WRKY46)	-3.3696 (down)	-1.4436 (down)	1.9278 (up)	AVESA.00010b.r2. 1CG0084810 (ERF034)	2.2722 (up)	3.83482 (up)	1.5641 (up)	AVESA.00010b.r2. 1AG0012840 (CYP98A1)	1.8264 (up)	3.1844 (up)	1.3573 (up)
AVESA.00010b.r2. 6DG1161830 (MYB39)	4.4278 (up)	3.2844 (up)	-1.1424 (down)	AVESA.00010b.r2. 7CG0703720 (WRKY57)	-3.5160 (down)	-1.4078 (down)	2.1099 (up)	AVESA.00010b.r2. 6CG1081480 (ERF1A)	-2.7508 (down)	-1.4676 (down)	1.2831 (up)	AVESA.00010b.r2. 3CG0466530 (CYP710A1)	2.5546 (up)	3.6824 (up)	1.1301 (up)
AVESA.00010b.r2. 1AG0045040 (MYB308)	4.1604 (up)	6.9409 (up)	2.7792 (up)	AVESA.00010b.r2. 2DG0381500 (WRKY71)	-4.2339 (down)	-1.2958 (down)	2.9374 (up)	AVESA.00010b.r2. 3AG0406380 (DREB1H)	-2.3650 (down)	1.3014 (up)	3.6664 (up)	AVESA.00010b.r2. 4CG1318300 (CYP714B2)	2.8208 (up)	1.4689 (up)	-1.3512 (down)

The values in the table are log_2_FC value between different comparison groups.

#### 3.4.5 DEGs clustering and expression profiling analysis

**Figure 7:**
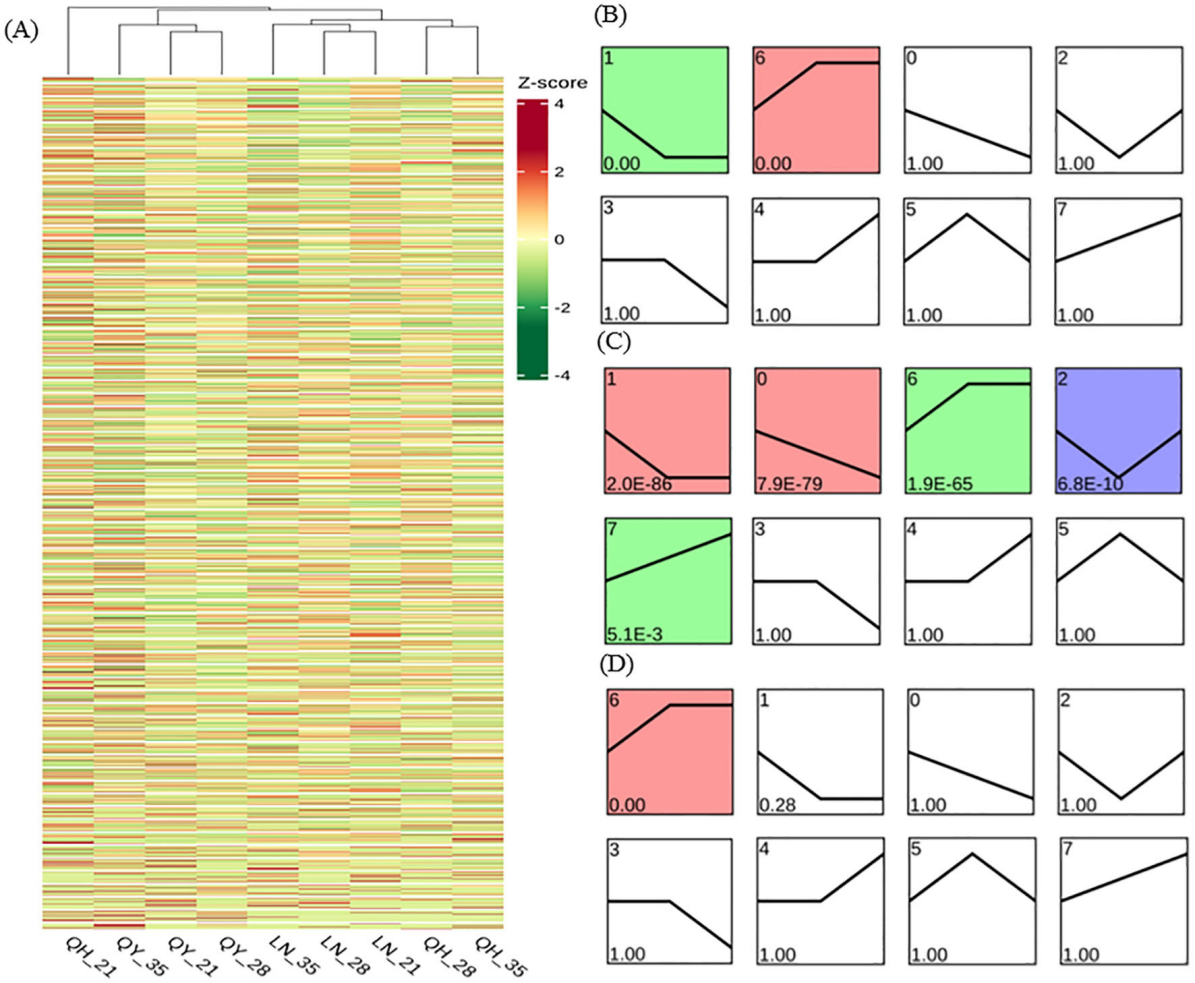
Gene expression trend analysis based on FPKM using log2 (fold change) hierarchical clustering analysis for DEG expression **(A)**, LENA **(B)**, Qingyin No.1 **(C)**, and Qingyin No.2 **(D)** at different time points during the growth in the second stem internode at the base of oats. The numbers in the upper left corner of the box represent different gene expression trend patterns. Numbers below the box are the p-values.

Through DEG cluster analysis, we found that there were significant differences in the expression of each gene in different materials and/or different growth stages (**Figure 7A**). In order to further study the gene expression profiles, the DEGs from the different growth stages were clustered into 8 Profiles by STEM software. Our results showed that Profile 1 and 6 were significantly expressed in ‘LENA’ (*P<* 0.05). Profile1 gene expression continuously decreased on the 21st and 28th day of growth, but then remained stable on the 35th day. Profile 6 showed that gene expression continuously increased on the 21st and 28th day of the growth, but then remained stable on the 35th day (**Figure 7B**).

In addition to Profile 1 and 6, Profile 0, 2 and 7 were also significantly expressed in ‘Qingyin No.1’ (*P*< 0.05). Profile 0 indicated that gene expression decreased during the different growth stages; Profile 2 pattern indicates that gene expression levels show a trend of first decreasing and then increasing at different growth stages of the second stem internode at the base of oats; Profile 7 gene expression showed an upward trend during the growth stages (**Figure 7C**). One major difference we noticed was that only Profile 6 were significantly expressed in ‘Qingyin No.2’ (*P*< 0.05), while Profile 1 were not enriched (**Figure 7D**). Our comprehensive analysis also showed that more than 50% of the DEGs were enriched into these five gene expression trend patterns.

#### 3.4.6 Screening and expression level analysis of candidate genes related to lodging resistance in oats

**Figure 8:**
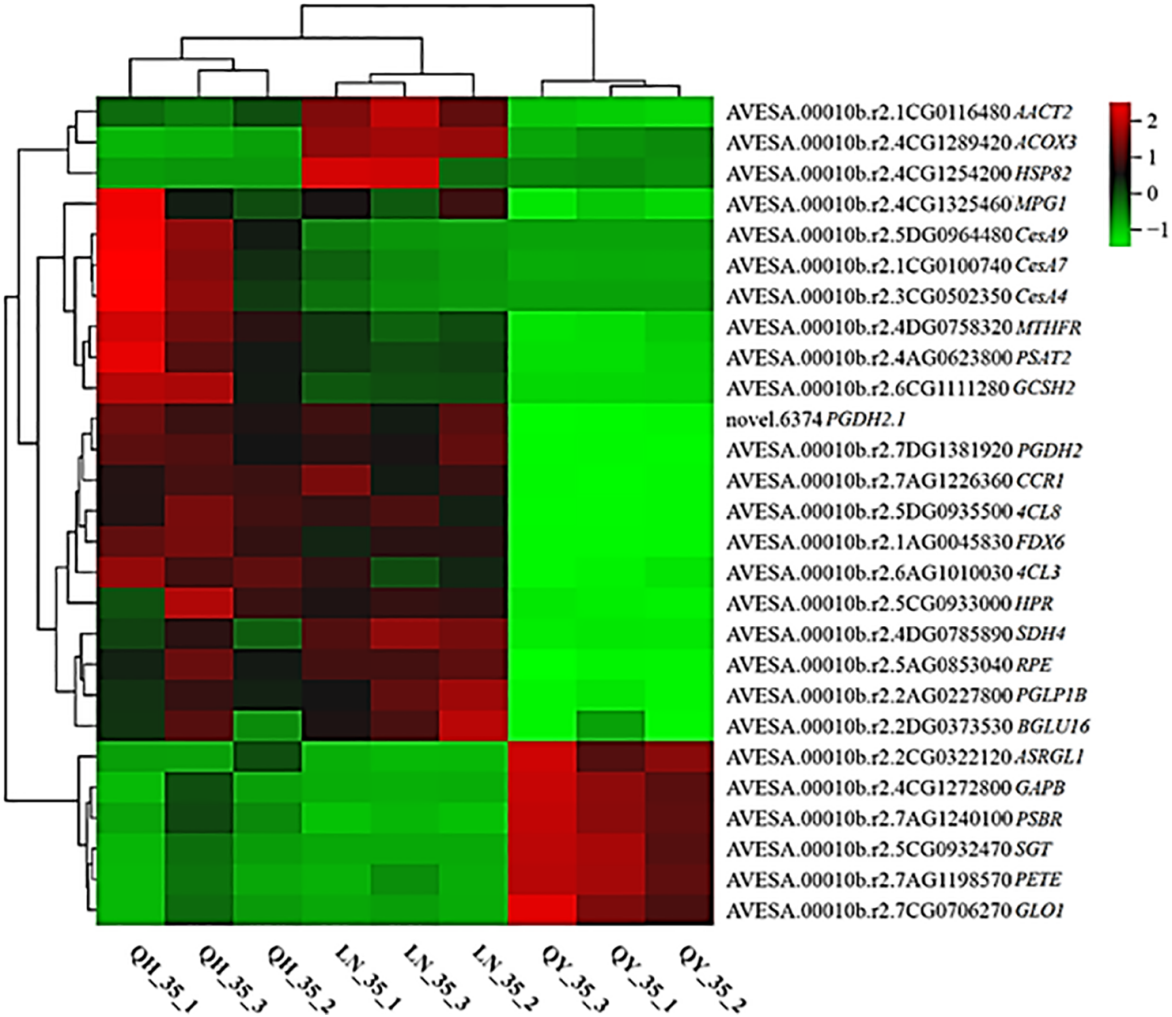
Candidate genes related to lodging resistance in oats.

Based on the results of venn diagram analysis, we found that the three oat cultivars had the most co-expressed genes on the 35th day of the growth of the second internode at the base. Therefore, we used the DEGs in this period to screen samples with the condition |log2Fold Change| ≥ 2 and FDR< 0.05, according to the expression level of DEGs and the results of KEGG function enrichment analysis. Our genetic screen found genes related to oat lodging resistance that involved photosynthesis (*FDX6*, *PSBR* and *PETE*), plant-pathogen interaction (*HSP82*), carbon metabolism (*GCSH2*, *SGT*, *SDH4*, *RPE*, *GAPB*, *PGDH2*, *PGDH2.1*, *AACT2*, *HPR*, *PSAT2*, *GLO1*, *PGLP1B*, *ACOX3* and *MTHFR*), cyanoamino acid metabolism (*ASRGL1* and *BGLU16*), amino sugar and nucleotide sugar metabolism (*MPG1*), phenylpropanoid biosynthesis (*CCR1* and *4CL3*) and glyoxylate and dicarboxylate metabolism (*4CL8*), as well as lignin synthase(*CesA9*, *CesA7* and *CesA4*) (**Figure 8**).

**Figure 8** also shows that there are some differences in the expression levels of the above genes among different cultivars of oats. The expression of most genes were up-regulated in the high lodging-resistant cultivar ‘LENA’ and the middle lodging-resistant cultivar ‘Qingyin No.1’. Among them, three key genes coding for enzymes (*CCR1*, *4CL8* and *4CL3*) were found to affect the content and composition of lignin in varying degrees, and played an important role in regulating crops lodging resistance. In addition, *CesA9*, *CesA7* and *CesA4* were specifically up-regulated in the middle lodging-resistant cultivar ‘Qingyin No.1’. These three genes are genes encoding enzymes related to cellulose synthesis, which played an important role in the formation of plant cell wall and can improve the mechanical support and resistance to environmental stress of plants. Meanwhile, *AACT2* and *ACOX3* were specifically up-regulated in the high lodging-resistant cultivar ‘LENA’, which suggested that these genes might play a role in improving lodging resistance in oats. Therefore, *CCR1*, *4CL8* and *4CL3*, as well as *CesA9*, *CesA7*, *CesA4*, *AACT2* and *ACOX3*, which are specific and highly expressed in high lodging-resistant and medium lodging-resistant cultivars, were selected as candidate genes related to lodging resistance for further experiments.

#### 3.4.7 Construction and module identification of weighted gene co-expression network

Our WGCNA analysis found that when the fitting curve exceeds 0.8, the soft threshold *β* = 8 can be determined (**Supplementary Figure S2A**). This integrated expression matrix was used to screen 34937 genes, and the dynamic tree cutting method was employed to select the optimal soft threshold *β* = 8 to construct gene co-expression network for oat lodging resistance. The network were constructed by filtered probes, and a total of 26 modules were identified. In these modules, the turquoise module had the largest number of DEGs (7573), while the dark-orange module had the least (39). Agrey module is a set of genes that are not assigned to other modules (**Supplementary Figure S2B**).

**Figure 9:**
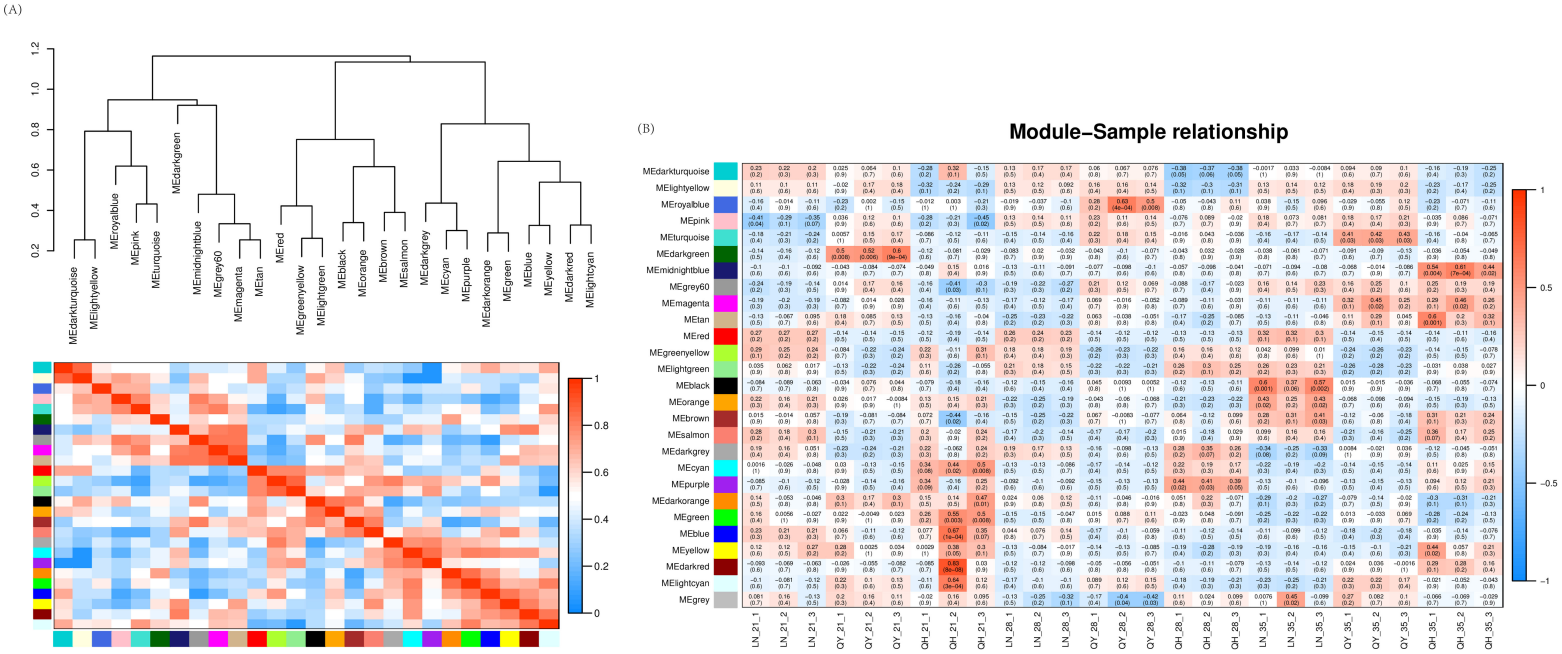
Clustering and heatmap analysis between modules **(A)**, with the clustering tree between modules above and the heatmap and modules below. Correlation heatmap **(B)** between modules and the second stem internode of three oat cultivars at different growth stages. The numbers in the figure represent the correlation between modules and samples, while the numbers in parentheses indicate the p-value of the correlation. The more red the heatmap, the more positively correlated it is. The more blue the heatmap, the more negatively correlated it is.

Through clustering and heat map analysis between the modules, we found that 26 modules are clustered in three branches, and that the relationships between the various branches are relatively close (**Figure 9A**). In the gene co-expression network of oat lodging resistance, the correlation coefficients between module characteristic genes and samples were tested to explore the relationship between the identified modules and oat lodging resistance traits. Among the 26 modules, only 4 modules were significantly correlated with lodging resistance in oats. Both the dark-red module and blue module were positively correlated with the 21st day of the growth in ‘Qingyin No.1’ (r^2^ = 0.83, *p* = 8e-08; r^2^ = 0.67, *p* = 1e-04). In addition, there was a significant positive correlation between the midnight-blue module and the 35th day of the growth and ‘Qingyin No.1’ (r^2^ = 0.61, *p* = 7e-04). Likewise, there was a significant positive correlation between the black module and the 35th day of the growth in the ‘LENA’ cultivar (r^2^ = 0.60, *p* = 0.001) (**Figure 9B**). By comprehensively considering the correlation between the modules and the correlation between module characteristic genes and samples, the blue, black, midnight-blue and dark-red module were selected as the key modules related to lodging resistance in oats that can be further mined and analyzed.

#### 3.4.8 GO and KEGG enrichment analysis of genes in each module

**Figure 10:**
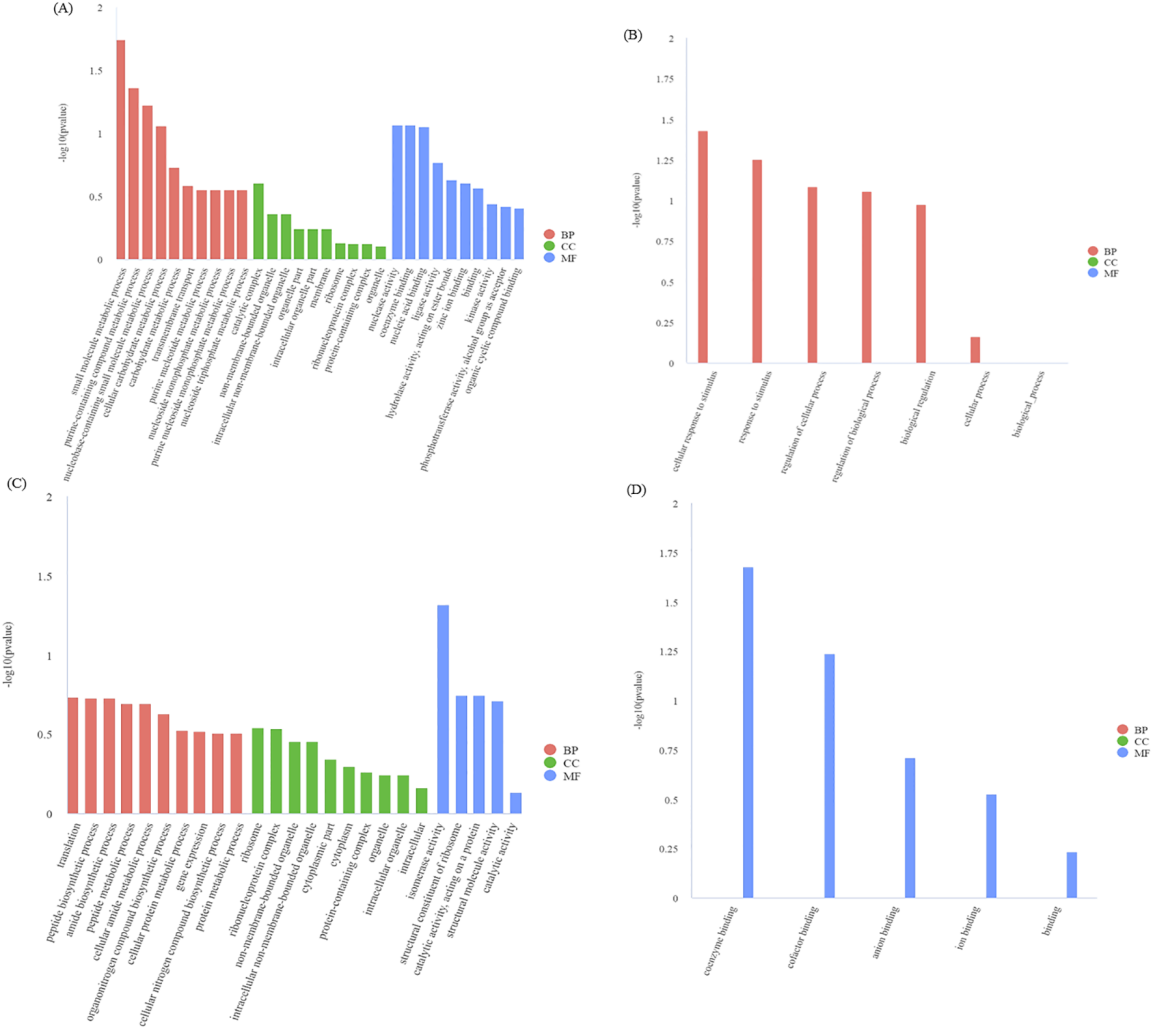
GO enrichment analysis of DEGs in the blue module **(A)**, black module **(B)**, midnight-blue module **(C)**, and dark-red module **(D)**. BP, biological process; CC, cellular component; MF, molecular function. The smaller the q-value, the higher the degree of enrichment. Same as below.

From our module data, the GO terms significantly enriched in genes in the blue module are GO: 0044281 (small molecular metabolic process) and GO: 0072521 (purine containing compound metabolic process) (**Figure 10A**). The GO terms with significantly enriched genes in the black module are GO: 0051716 (cellular response to stimulus) and GO: 0050896 (response to stimulus) (**Figure 10B**). The GO terms enriched in genes in the midnightblue module are GO: 0006412 (translation) and GO: 0043043 (peptide biosynthetic process) (**Figure 10C**). Finally, the GO terms enriched in the darkred module are GO: 0050662 (coenzyme binding) and GO: 0048037 (cofactor binding), etc (**Figure 10D**).

**Figure 11:**
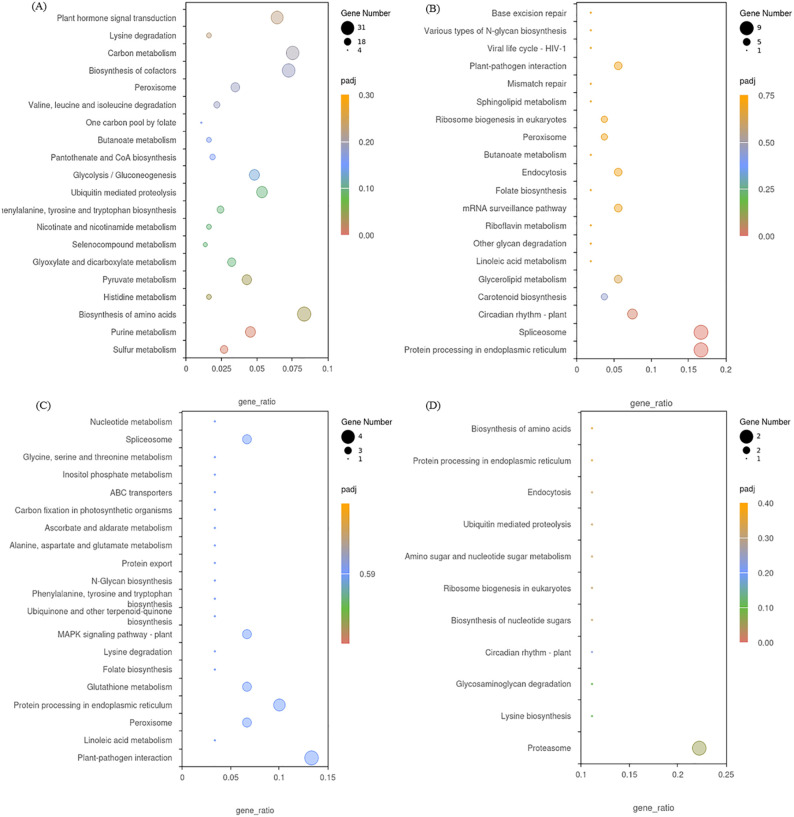
KEGG enrichment analysis of DEGs in the blue module **(A)**, black module **(B)**, midnight-blue module **(C)**, and dark-red module **(D)**.

Our KEGG pathway analysis showed that 112, 49, 32 and 11 enriched KEGG pathways were identified by from the DEGs in the four modules. DEGs in the blue module were significantly enriched in sulfur metabolism and purine metabolism (**Figure 11A**), while DEGs in the black module were significantly enriched in protein processing in endoplasmic reticulum and spliceosome (**Figure 11B**). DEGs in the midnight-blue module were mainly enriched in plant-pathogen interaction and linoleic acid metabolism (**Figure 11C**), while DEGs in the dark-red module were significantly enriched in proteasome and lysine biosynthesis (**Figure 11D**).

#### 3.4.9 Screening and expression analysis of candidate hub genes

**Figure 12:**
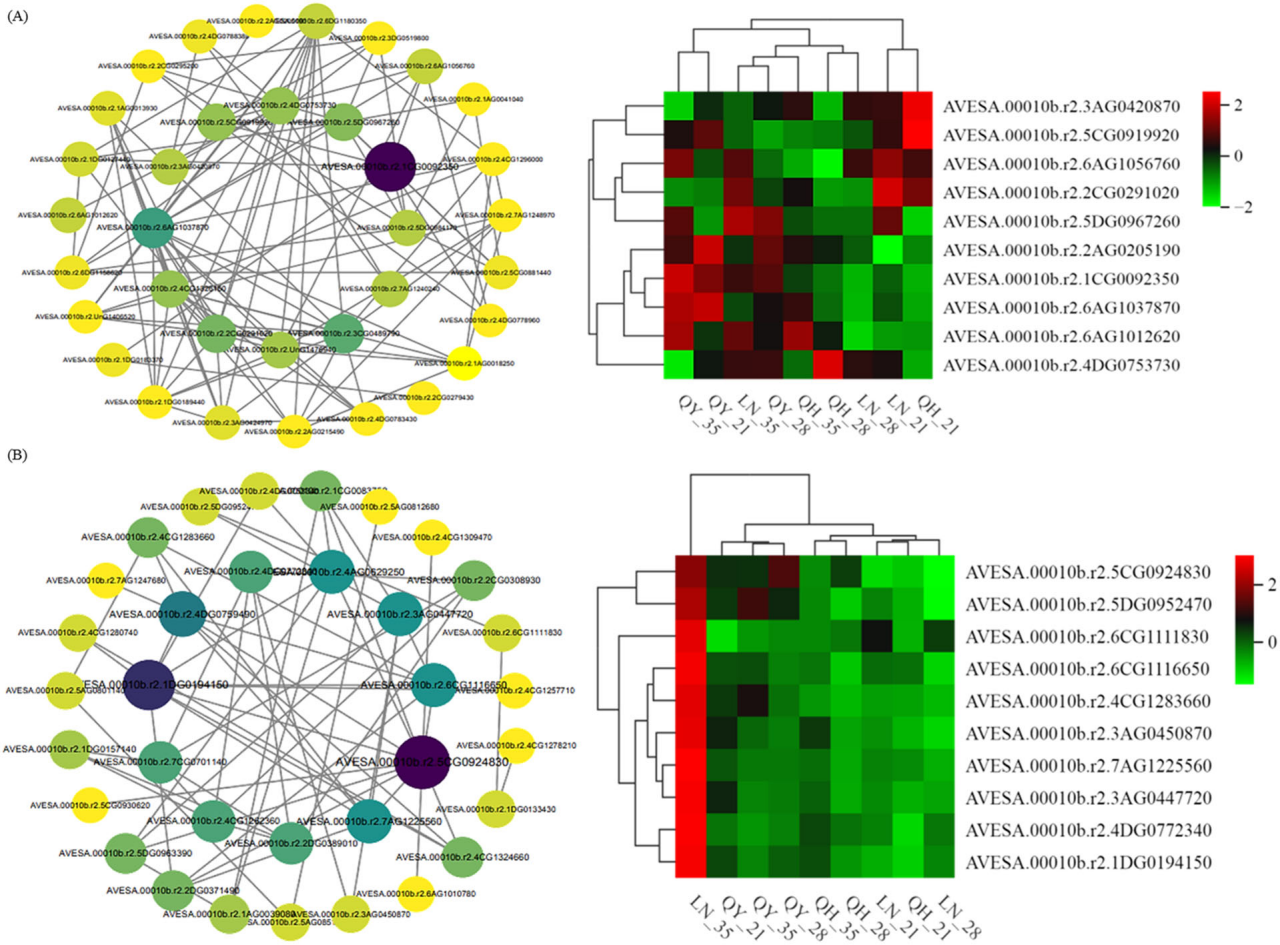
The co-expression network of genes in the blue **(A)** and black module **(B)**. The expression pattern of Hub genes were analyzed. The central circle represents the Hub genes and the genes with high connectivity in the corresponding network are shown in the larger circle.

In total, 20 genes with high connectivity in blue and black modules were identified as central genes (**Figures 12A, B**), while midnight-blue and dark-red modules were not built into the PPI network. Genes such as 30s ribosomal protein S13 (AVESA.00010b.r2.5DG0967260, *RPS13*), citrate synthase 4 (AVESA.00010b.r2.6AG1012620, *CS4*) and DNA-directed RNA polymerases II, IV and V subunit 9A (AVESA.00010b.r2.6AG1056760, *RPB9*) in blue module may be related to plant lodging resistance. In addition, the expression of the *RPS13* gene is the highest on the 35th day of basal second stem internode growth in the high lodging-resistant cultivar ‘LENA’, while the expression of *CS4* gene was the highest on the 35th for the middle lodging-resistant cultivar ‘Qingyin No.1’. Furthermore, the expression of *RPB9* gene was the highest on the 21st day of the growth for the high lodging-resistant cultivar ‘LENA’ (**Figure 12A**).

Genes in the black module, such as multiprotein-bridging factor 1c (AVESA.00010b.r2.4CG1283660, *MBF1c*), SKP1-like protein 1B (AVESA.00010b.r2.5DG0952470, *SKP1*), and cullin-associated NEDD8-dissociated protein 1 (AVESA.00010b.r2.6CG1111830, *CAND1*) were highly expressed on the 35th day after the growth in the high lodging-resistant cultivar ‘LENA’ (**Figure 12B**), which were also previously speculated to have a certain regulatory effect on the lodging resistance of oats.

## 4 Discussion

### 4.1 Study on the relationship between phenotypic morphological characteristics and lodging resistance in oats

Plant lodging decreases quality and mechanical harvest efficiency, as well as increases grain dry rate by as much as 20%, which has a serious impact on production efficiency, and has become a key problem that restricts grain production (Wen et al., 2019). Oat is one of the main cultivars in the Qinghai-Tibet Plateau, the climatic conditions and soil types in this area are suitable for the growth of oats, but during the growth and development process of oats in high-altitude regions, lodging is an inevitable phenomenon, this has had a great impact on the development of animal husbandry in high-altitude pastoral areas. Plant phenotypic morphological characteristics are one of the most important traits that determine crop lodging resistance, and it is known as the most intuitive and easily identifiable feature to determine lodging. The stem is the main supporting organ for plant growth and development, and its internode length, stem thickness, and stem wall thickness are closely related to the lodging condition of the plant. It has been shown that plant lodging is mainly related to plant height and center of gravity height (Ahmad et al., 2023), and the cultivars with large height of stem center of gravity, unbalanced allocation of each internode, long basal second internode and large proportion of ear height, but has thin stems, thin wall, low density and plumpness had poor stem quality, low folding resistance and large lodging index. Lodging is prone to occur in the field, which is similar to the results of this study, which found that the phenotypic morphological characteristics of oat stem are closely related to its lodging resistance, and there is a significant positive correlation between the lodging index of the second stem internode and the actual lodging rate at flowering stage, the higher the value of both, the weaker the ability to resist lodging. The lodging index of the second stem internode showed a significant positive correlation with plant height, the length of the second stem internode, the height of ear height and the coefficient of ear position, and a significant negative correlation with the stem wall thickness of the second stem internode. This shows that the larger the plant height and the length of the second stem internode, the more prone to lodging, and increasing the stem wall thickness of the second stem internode will reduce the risk of oat lodging.

In addition, the stem internode composition of the stem was most closely related to the lodging resistance of the first and second internode of the main stem and the subpanicle internode. The structural characteristics, coordination degree and allocation ratio of the basal internodes jointly determined the lodging resistance of oat plants (Nan, 2021). In particular, Liu (2005) found that cultivars with strong lodging resistance had short basal internodes, thick culm walls, longer flag leaves, smaller leaf area, higher mechanical strength, and greater elasticity in their stems. This study found that there was a significant positive correlation between the actual lodging rate of oat and the length of the second stem internode, and a significant negative correlation between the second stem internode lodging index and compression strength, puncture strength, breaking force and mechanical strength. The second stem internode length of ‘Qingyin No.2’ was higher than that of ‘LENA’ and ‘Qingyin No.1’. The compression strength, puncture strength, breaking force and mechanical strength of ‘LENA’ and ‘Qingyin No.1’ were higher than those of ‘Qingyin No.2’, which were similar to that of Liu (2005), indicated that the oat cultivars with strong lodging resistance had shorter basal internodes and higher mechanical strength and compression strength. Nan (2021) found that the oat cultivars with short internodes, thick culm walls, more lignin in the cell wall, high hardness and plumpness in the cell wall, as well as a small pulp cavity volume created a strong resistance to lodging with high stem quality. Liang et al. (2019) also found that the stem diameter, stem diameter coefficient, and internode length of the second stem internode at the base of oat stem were significantly higher than those of the first stem internode. However, the stem wall thickness and mechanical properties of the first stem internode were significantly higher than those of the second stem internode, and stem base traits were the main factors affecting oat lodging. Based on previous analysis, this study found that oat lodging mainly occurs at the second stem internode at the base, the actual lodging rate of the second stem internode were significantly positively correlated with the length of the second stem internode, and significantly negatively correlated with the stem thickness, stem wall thickness, compression strength, puncture strength, breaking force, and mechanical strength of the second stem internode, this is similar to the results of Nan (2021) and Liang et al. (2019). Oats with strong lodging resistance have lower plant height and height of center of gravity, base stem thick and stem wall thick, shorter internodes, and better mechanical properties, these traits collectively affect the lodging resistance of oats. Based on these results, subsequent lodging studies in oat can focus on the second stem internode at the base to enhance the strength and toughness of the stem internode, and improve the lodging resistance. Plant height should also be investigated in lodging research, since the lodging resistance of oats can be increased by reducing plant height.

### 4.2 Study on the relationship between physicochemical properties and lodging resistance in oats

The physicochemical properties of plant stems are closely related to their lodging resistance. If some or a certain component in the stems is out of proportion or physiological disorders occur, it will increase the risk of lodging. Generally, the most abundant substances in the stem include trace mineral elements, lignin, cellulose, polysaccharides, and other non-structural carbohydrates, which mainly exist in the thin-walled cells of the stem. These substances coordinate and maintain the “source-sink” at different stages of plant growth and development, which affect the process of grain filling. Fan et al. (2017) found that the content of mineral elements, cellulose, and lignin in rice stem tissue cells maintains stable stem mechanical properties and support strength. They also found that increasing cellulose and lignin content improves stem mechanical strength and surface cell wall hardness to support the cell wall structure, which is more conducive to enhancing lodging resistance. This study found that the physiological indicators of the stems of three different lodging-resistant oat cultivars were extremely significant/significantly negative correlated with the lodging index and actual lodging rate of the second stem internode during flowering. Analysis of their lignin content revealed that the lodging index and actual lodging rate of the second stem internode during flowering were significantly negatively correlated with lignin content. The lignin content in the stems of lodging-resistant cultivars was higher than that of lodging susceptible cultivar, which is similar to the research results of Fan et al. (2017), indicating that higher lignin content can enhance the mechanical strength and epidermal cell wall hardness of oat stems, thereby reducing the risk of lodging.

Nan (2021) also found that the amount of potassium, calcium, silicon, and magnesium in the basal internodes of the resistant cultivars were significantly higher than those of the lodging susceptible cultivar. Low nitrogen and high potassium, calcium, silicon, and magnesium content are the key to enhance the lodging resistance in oat stems, which is similar to the results of this study. This study found that the lodging index of the second stem internode during oat flowering were extremely significant negative correlated with stem calcium and silicon content, and significantly negatively correlated with potassium content, the actual lodging rate during the flowering period were extremely significant negative correlated with stem silicon content, and significantly negatively correlated with potassium and calcium content. This indicating that the higher the content of potassium, calcium and silicon in oat stem, the stronger the lodging resistance, and the higher the content of potassium, calcium and silicon will enhance the mechanical strength and toughness of oat stem, make the stem harder, thus enhance the lodging resistance.

In addition, non-structural carbohydrates are the main enrichment substances in crop stems, especially polysaccharides such as starch and soluble sugars, which can promote the formation of cellulose and hemicellulose, thereby helping to maintain the mechanical strength of the stem. Zhang et al. (2010) found in their study on rice that the soluble sugar content in the stem and sheath in lodging-resistant cultivars was higher during the late flowering stage. However, there was not a correlation between soluble sugar content in the stem and lodging resistance during the milky stage. Ishimaru et al. (2008) also showed that the rice stem was easily bent by the wind, and that the cultivars with higher starch content in the stem were more beneficial to return to the normal growth state. Zhang et al. (2020) found that starch content were significantly positively correlated with cellulose content and hemicellulose content, which suggests that higher starch content in oat stems could promote the synthesis of cellulose and hemicellulose to improve their lodging resistance, which is similar to the results of this study. In this paper, based on the analysis of soluble sugar and starch content in oat stem, it was found that the lodging index and actual lodging rate of the second stem internode at flowering stage were extremely significant negative correlated with stem soluble sugar and starch content, and the soluble sugar and starch content in plant stem could affect the bending strength of oat stalk. This shows that increasing the content of soluble sugar and starch in oat stem can effectively enhance the lodging resistance of the species.

### 4.3 Regulation of transcription factors on lodging resistance in oat stalk

As an important regulatory protein, TFs specifically bind to cis-acting elements in the promoter region of eukaryotic genes to regulate the expression of target genes. They also played a central role in regulatory networks and signaling pathways during plant development, as well as respond to abiotic stress (Cui et al., 2016). Li et al. (2024) found that NAC and MYB family can regulate the development of plant secondary walls, especially the synthesis and accumulation of lignin, playing an important regulatory role in plant secondary growth and lodging resistance. Xiao et al. (2018) found that NAC can enhance *CesA* gene expression by promoting MYB transcription to enhance cellulose synthesis, and improve the mechanical strength and toughness of maize (*Zea mays*) stalks. This study found that TFs of the MYB family account for a relatively large proportion in the growth of the second stem internode at the base of different oat cultivars, which is consistent with previous research results. In addition, Wang et al. (2024) found that MYB110 can function to regulate plant height and lodging resistance, since MYB110 is the target gene of OsPHR2, which is the core regulator in the rice phosphorus signal transduction network. Here, they found lodging resistance was positively regulated by OsPHR2, which suppresses rice plant height. Although they saw plant height increased and stem lignin content decreased, their myb110 mutants had stronger lodging resistance since their stems were stronger than wild-type plants. In this study, we found that TFs such as *MYB86*, *ODORANT1*, *MYBHv33*, *MYB308*, and *MYB39* were significantly up-regulated in lodging-resistant cultivar. It is speculated that these TFs may also affect plant height and stem strength by regulating cell division and differentiation, thereby regulating the synthesis of cellulose and lignin and affecting the stiffness and toughness of oat stems, directly affecting the lodging resistance of oats. Besides, Lv et al. (2024) showed that *OsTCP19* of the TCP TF family can regulate the synthesis of cellulose and lignin in rice, thereby affecting the stiffness and toughness of stems. Our study also found significant differences in a small number of TCP TFs among different lodging-resistant oat cultivars, similar to previous research results.

Cytochrome P450 (CYP450) catalyzes the 3-hydroxylation of phenylpropane compounds to modulate the precursors of secondary metabolites like lignin and anthocyanins, which may affect the lignin content in plant stems and thus stem strength (Morant et al., 2007). Kim et al. (2005) found that most *cyp85a* mutants in plants exhibit a super compact phenotype with shortened internodes that lead to dwarfism and a reduced risk of lodging. Our study also discovered a family of cytochrome P450 TFs, including *CYP89A9*, *CYP75A5*, *CYP98A1*, *CYP710A1*, and *CYP714B2*, which were up-regulated during the growth of the second stem internode of the high lodging-resistant oat ‘LENA’. And the plant height of ‘LENA’ was significantly lower than the other two oat varieties, suggesting that these TFs may improve its lodging resistance by regulating the content of enriched substances in the ‘LENA’ stem and shortening internode length.

WRKY family can affect secondary cell wall thickening by binding to the promoters of other TFs that activate secondary wall synthesis (Wang et al., 2010). Lan et al. (2020) also showed that *WRKY36* inhibits gibberellins (GAs) signaling by stabilizing the expression of the GAs inhibitor *SLR1* to decrease rice grain size and plant height and reduce the occurrence of lodging. Similarly, multiple WRKY transcription factors were found to be differentially expressed during the growth process of the second stem internode in different lodging-resistant oat cultivars. Among them, the expression of *WRKY6*, *WRKY46*, *WRKY57*, and *WRKY71* were down-regulated in lodging-resistant cultivars, while *WRKY22* was up-regulated. It is speculated that these TFs can reduce the risk of oats lodging by regulating the thickness of the secondary cell wall of oat stems or reducing their plant height. AP2/ERF TF family played an important role in plant growth and development and stress response. Du (2021) and Qi et al. (2011) found that *OsRPH1* and *OsRPH2* of the AP2/ERF family can regulate the expression levels of genes related to GA synthesis metabolism, which reduce endogenous GA activity in rice and negatively regulate plant height to improve the lodging resistance and yield of rice. In this study, it was found that the TFs of the AP2 family also account for a large proportion in comparison groups of oats. Among them, the expression of *ERF055*, *DREB3*, and *ERF034* were up-regulated in lodging-resistant oats. It is speculated that these TFs may have similar roles in regulating oat lodging resistance.

### 4.4 Study on genes related to lodging resistance in oats

In recent years, high-throughput sequencing technology (RNA-seq) has rapidly developed, which can effectively understand the regulatory rules of gene expression, and identify key genes related to plant function regulation (Qiong et al., 2019). Liu et al. (2022) studied the lodging resistance traits of maize by integrating transcriptome and metabolome analysis, KEGG enrichment analysis found that DEGs were mainly enriched in phenylpropanoid and secondary metabolites biosynthesis pathways, and several functional and regulatory genes related to lodging resistance traits were screened, these genes mainly participate in cell wall assembly, lignin biosynthetic process, and hormone metabolic process. In present study, transcriptome was conducted on the different lodging-resistant oat cultivars and found that the three oat cultivars co-expressed the most genes on the 35th day of growth between the second stem internodes at the base, and these DEGs were significantly enriched in pathways such as starch and sucrose metabolism, phenylpropanoid biosynthesis, MAPK signaling pathway-plant and carbon metabolism.Genes related to cellulose synthesis and lignin synthesis were found in these pathways, which is similar to previous research results. Among them, the cellulose synthesis genes *CesA4*, *CesA7*, and *CesA9* are specifically up-regulated in the middle lodging-resistant cultivar ‘Qingyin No.1’. Studies have shown that the expression of *CesA4*, *CesA7*, and *CesA9* genes in constitutes the synthase complex necessary for the synthesis of secondary cell wall cellulose (Maleki et al., 2016). Li et al. (2018) found in their study on rice that mutations at the *CESA4* P-CR site can affect cell wall characteristics, particularly cellulose structure, thereby enhancing biomass digestion and lodging resistance; Kumar et al. (2017) and Deng et al. (2019) also showed that a deletion mutant in *cesa7* decreased cellulose content in the stem and created defects in secondary cell wall formation in the xylem, which led to the collapse of xylem constituent elements. Li et al. (2017) also found that mutant *OsCesA9* reduced plant height and showed thinner stems with significantly increased tiller numbers compared to the wild type. It was speculated that high lodging-resistant in this mutant may be attributed to its plant height, single tiller fresh weight, and low cellulose crystallinity. These results indicate that different genes in the *CesA* family related to cellulose synthesis contribute to cellulose synthesis in primary and secondary cell walls during plant development, thereby enhancing plant lodging resistance. Therefore, we speculate that these three genes are closely related to the lodging resistance characteristics of oat stems.

In addition, lignin enhances the mechanical strength of the stem by participating in cell wall biosynthesis to improve the lodging resistance in crops. Hu et al. (2015) showed that the lignin biosynthesis related gene *4CL* in the stem of *Fagopyrum esculentum* reached its highest expression during the full flowering stage, and that the expression level in lodging-resistant cultivar were significantly higher than that in lodging-susceptible cultivar, this is consistent with the research findings of this paper. In this paper, *4CL8* and *4CL3* genes related to lignin synthesis were screened, these two genes were specifically up-regulated in high lodging-resistant cultivar ‘LENA’ and middle lodging-resistant cultivar ‘Qingyin No.1’. The expression level in lodging-resistant cultivars were significantly higher than that in lodging-susceptible cultivar, which indicated that lignin synthesis related genes played an important role in the regulation of crops lodging resistance. CCR is the starting point in the special branching pathway for the synthesis of lignin in the phenylpropane metabolic pathway. Here, it acts as a key enzyme to control the content and quality of lignin, and changing *CCR* gene expression levels will affect the total lignin content in plants (Meester et al., 2020). In this paper, another gene related to lignin synthesis, *CCR1*, was also screened, which was specifically up-regulated in lodging- resistant cultivars, and the expression level in lodging-resistant cultivar were significantly higher than that in lodging-susceptible cultivar, Zhou et al. (2010) found that the growth rate and lignin content of *Medicago truncatula* decreased significantly after inhibiting the expression of *CCR1*; Hu et al. (2017) found that the lodging resistance of buckwheat increases with the relative expression levels of genes such as *PAL*, *4CL*, *C4H*, *CAD*, and *CCR*, this indicates that up-regulated of lignin synthesis related genes in plants promotes an increase in lignin content, thereby enhancing the lodging resistance of plants. In addition, in the high lodging-resistant cultivar ‘LENA’, *AACT2* and *ACOX3* were specifically up-regulated, suggesting that these genes may play a certain role in improving the lodging resistance of oats.

WGCNA analysis based on large sample transcriptome data has become the most widely used co-expression network analysis method, not only for constructing gene networks, but also for detecting hub genes in gene modules and identifying them (Langfelder and Horvath, 2008). It will indicate the similarity of expression patterns between genes and whether they are in the same expression module, which is suitable for complex multi sample transcriptome data. It can quickly study the overall expression pattern of genes and provide feedback on the interaction patterns between genes in the sample (Zhang and Horvath, 2005). At present, the WGCNA analysis method has been widely used in the screening of stress resistance related genes in various plants. Chen et al. (2022) conducted WGCNA analysis on transcriptome data sequenced from cotton (*Gossypium hirsutum*) under salt and drought stress, and identified that *GhWRKY46* gene can improve drought and salt tolerance of cotton. Wang et al. (2023) used WGCNA to construct a gene co-expression network and identified 20 hormone related candidate genes associated with salt stress in tomato (*Solanum lycopersicum*). In this study, the hub genes closely related to the lodging resistance traits of oats, such as *MBF1c*, *SKP1* and *CAND1*, were screened by WGCNA analysis, and it was found that they were specifically expressed on the 35th day of internode growth of the second stem at the base of the lodging resistance cultivar ‘LENA’. Among them, *MBF1c* is a transcription co-activator that has been shown to affect the stem stiffness and toughness of plants by regulating the synthesis of plant cell wall components (Liu et al., 2023), which is consistent with the KEGG enrichment pathway results; In addition, the protein encoded by *SKP1* can bind to Cullin protein and F-box protein, forming the core part of SCF complex, enhanced expression of this gene can improve plant resistance (Farrás et al., 2001); CAND1 is a protein associated with NEDD8 modification, and the *SlCAND1*-RNAi line were significantly dwarfed compared with wild-type plants during the vegetative growth stage of tomato, which effectively reduced the risk of lodging (Cheng et al., 2020). Based on phenotype data, we found that the lodging-resistant cultivar ‘LENA’ has lower plant height and stronger lodging resistance, and the high expression of this gene in ‘LENA’ is further confirmed the efficiency and accuracy of the WGCNA analysis method. This study comprehensively utilized transcriptome analysis and WGCNA analysis to obtain multiple genes related to oat lodging resistance, providing important genetic resources for the breeding of lodging-resistant oat cultivars. At present, molecular marker assisted breeding and CRISPR-Cas9 gene editing technology have brought revolutionary changes to biological breeding, they can accurately locate and modify target genes, thereby quickly cultivating crop cultivars with excellent traits. Applying these genes to these cutting-edge technologies is expected to open up new avenues for cultivating new cultivars of lodging-resistant oats and promote innovative development in oat breeding.

## 5 Conclusion

Our study conducted transcriptome analysis on the field traits and stem physiological indicators of three oat cultivars with different lodging resistance abilities during the key period of growth for the second stem internode at the base. We found that the lodging resistance of oats was closely related to the changes in stem growth characteristics and physicochemical component content. On the 35th day of growth, there are more co-expressed genes, which indicates that the expression of regulatory pathways may become more complex. The growth process of the second stem internode at the base of this species is mainly related to the KEGG pathway, such as starch and sucrose metabolism, phenylpropanoid biosynthesis, MAPK signaling pathway-plant and carbon metabolism. TFs from families like p450, Myb_DNA-binding, WRKY, and AP2, along with cellulose synthesis related genes *CesA9*, *CesA7*, and *CesA4*, lignin synthesis related genes *CCR1*, *4CL8*, and *4CL3*, as well as genes *AACT2*, *ACOX3*, *MBF1c*, *SKP1*, and *CAND1* that are specifically up-regulated in the high lodging-resistant cultivar ‘LENA’, jointly regulate the growth process of the second stem internode at the base of oats. It is speculated that these TFs and genes have a certain regulatory effect on the lodging resistance of oats. Further research on the functions of these genes will help reveal the molecular mechanisms of oat lodging resistance and provide excellent genetic resources for lodging resistance breeding in oats and other crops.

## Data Availability

The datasets presented in this study can be found in online repositories. The names of the repository/repositories and accession number(s) can be found below: https://www.ncbi.nlm.nih.gov/, PRJNA1186259.
