# Supplementary material for: Physiological mechanism of lodging resistance of oat stalk and analysis of transcriptome differences
Source: Front Plant Sci. 2025 Apr 2;16:1532216. doi: 10.3389/fpls.2025.1532216 (PMC12000040; doi:10.3389/fpls.2025.1532216)
Supplement: Supplementary file 1 [file DataSheet1.docx]

Supplementary Material

# Supplementary Figures and Tables

## Supplementary Figures


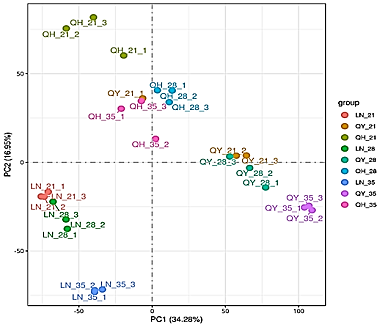


**Supplementary** **Figure 1.** Principal component analysis of different oat cultivars. PC1 represents the first principal component and PC2 represents the second principal component.

**
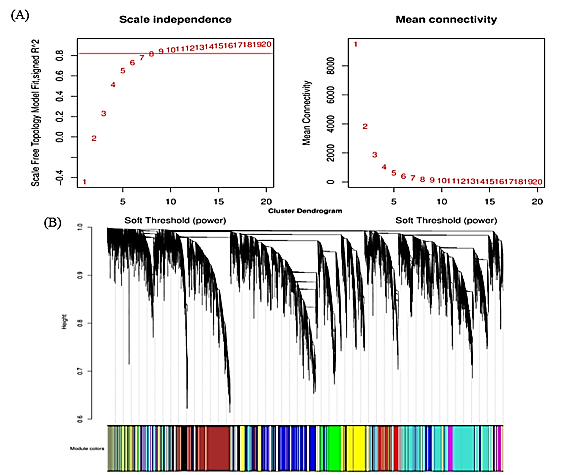
**

**Supplementary Figure 2.** Soft threshold network topology analysis(A), the left panel shows the scale-free fitting index (y-axis) as a function of the soft threshold (x-axis), and the right panel shows the average connectivity (y-axis) as a function of the soft threshold (x-axis); Gene clustering tree and module segmentation (B), and the upper part of the graph is the stagger clustering tree of the sample; Module colors represents the module divided according to the expression of each gene.

## Supplementary Tables

# Supplementary Table 1. Quality information of sequencing data and comparative analysis with reference genome.

| Sample | Raw Reads | Raw bases | Clean Reads | Clean Bases | Error Rate(%) | Q20  (%) | Q30  (%) | GC Content(%) | total_map(%) |
| --- | --- | --- | --- | --- | --- | --- | --- | --- | --- |
| LN_21_1 | 70745190 | 10.61G | 68914452 | 10.34G | 0.02 | 98.45 | 95.45 | 54.33 | 66969837(97.18) |
| LN_21_2 | 72217738 | 10.83G | 71120156 | 10.67G | 0.02 | 98.19 | 94.90 | 54.41 | 68823857(96.77) |
| LN_21_3 | 69877462 | 10.48G | 68239700 | 10.24G | 0.02 | 98.31 | 95.13 | 54.52 | 66156013(96.95) |
| QH_21_1 | 68822704 | 10.32G | 68174766 | 10.23G | 0.02 | 98.30 | 95.20 | 54.80 | 67931585(95.72) |
| QH_21_2 | 69981470 | 10.5G | 69092992 | 10.36G | 0.02 | 98.32 | 95.23 | 55.75 | 69537853(95.43) |
| QH_21_3 | 72157994 | 10.82G | 71234306 | 10.69G | 0.02 | 98.27 | 95.03 | 55.15 | 60548973(95.00) |
| QY_21_1 | 71932584 | 10.79G | 70965842 | 10.64G | 0.02 | 98.28 | 95.10 | 55.11 | 65000797(95.34) |
| QY_21_2 | 73850094 | 11.08G | 72871174 | 10.93G | 0.02 | 98.22 | 94.65 | 54.82 | 65820107(95.26) |
| QY_21_3 | 64466448 | 9.67G | 63733178 | 9.56G | 0.02 | 98.02 | 94.50 | 54.88 | 67963436(95.41) |
| LN_28_1 | 81494552 | 12.22G | 80482734 | 12.07G | 0.02 | 98.24 | 95.02 | 54.29 | 77982432(96.89) |
| LN_28_2 | 65409164 | 9.81G | 64563008 | 9.68G | 0.02 | 98.18 | 94.88 | 54.64 | 62507032(96.82) |
| LN_28_3 | 71739200 | 10.76G | 70828842 | 10.62G | 0.02 | 98.27 | 95.10 | 55.25 | 68534518(96.76) |
| QH_28_1 | 71636828 | 10.75G | 70830666 | 10.62G | 0.02 | 98.37 | 95.25 | 54.33 | 68071773(95.15) |
| QH_28_2 | 79391648 | 11.91G | 78422768 | 11.76G | 0.02 | 98.26 | 95.04 | 54.48 | 70564301(95.18) |
| QH_28_3 | 77807174 | 11.67G | 76812366 | 11.52G | 0.02 | 98.34 | 95.17 | 53.89 | 69961412(95.38) |
| QY_28_1 | 72238884 | 10.84G | 71538296 | 10.73G | 0.02 | 98.21 | 94.97 | 54.86 | 67475699(95.26) |
| QY_28_2 | 75022738 | 11.25G | 74139162 | 11.12G | 0.02 | 98.36 | 95.28 | 55.11 | 74643385(95.18) |
| QY_28_3 | 74793866 | 11.22G | 73347732 | 11.00G | 0.02 | 98.40 | 95.41 | 55.17 | 73122211(95.20) |
| LN_35_1 | 77741594 | 11.66G | 77015880 | 11.55G | 0.02 | 98.21 | 94.80 | 54.28 | 74674415(96.96) |
| LN_35_2 | 70722074 | 10.61G | 69958074 | 10.49G | 0.02 | 98.20 | 94.90 | 53.87 | 67668813(96.73) |
| LN_35_3 | 82340082 | 12.35G | 81158616 | 12.17G | 0.02 | 98.23 | 95.00 | 54.28 | 78703816(96.98) |
| QH_35_1 | 80303372 | 12.05G | 79525562 | 11.93G | 0.02 | 98.26 | 95.03 | 54.64 | 66623608(94.91) |
| QH_35_2 | 68885154 | 10.33G | 68224876 | 10.23G | 0.02 | 98.12 | 94.45 | 54.57 | 79494120(95.27) |
| QH_35_3 | 70127992 | 10.52G | 69263034 | 10.39G | 0.02 | 98.19 | 94.88 | 54.31 | 67259255(94.88) |
| QY_35_1 | 71022184 | 10.65G | 70198634 | 10.53G | 0.02 | 98.21 | 94.90 | 54.24 | 75771950(95.28) |
| QY_35_2 | 84501446 | 12.68G | 83441748 | 12.52G | 0.02 | 98.38 | 95.32 | 54.53 | 65011777(95.29) |
| QY_35_3 | 71727118 | 10.76G | 70890898 | 10.63G | 0.02 | 98.27 | 95.10 | 54.32 | 66059313(95.37) |

# LN_21, The 21st day of growth of the second stem internode at the base of 'LENA'; QH_21, The 21st day of the growth of the second stem internode at the base of 'Qingyin No.1'; QY_21, The 21st day of the growth of the second stem internode at the base of 'Qingyin No.2'; LN_28, The 28th day of growth of the second stem internode at the base of 'LENA'; QH_28, The 28th day of the growth of the second stem internode at the base of 'Qingyin No.1'; QY_28, The 28th day of the growth of the second stem internode at the base of 'Qingyin No.2'; LN_35, The 35th day of growth of the second stem internode at the base of 'LENA'; QH_35, The 35th day of the growth of the second stem internode at the base of 'Qingyin No.1'; QY_35, The 35th day of the growth of the second stem internode at the base of 'Qingyin No.2'.
